# Inter-organelle communication dynamically orchestrates juvenile hormone biosynthesis and female reproduction

**DOI:** 10.1093/nsr/nwaf022

**Published:** 2025-01-23

**Authors:** Shiming Zhu, Fangfang Liu, Xiaoyi Chen, Sishi Xia, Yingting Wu, Wei Tang, Chonghua Ren, Jian Wang, Sheng Li

**Affiliations:** Guangdong Provincial Key Laboratory of Insect Developmental Biology and Applied Technology, Guangzhou Key Laboratory of Insect Development Regulation and Application Research, Institute of Insect Science and Technology, School of Life Sciences, South China Normal University, Guangzhou 510631, China; Guangmeiyuan R&D Center, Guangdong Provincial Key Laboratory of Insect Developmental Biology and Applied Technology, South China Normal University, Meizhou 514779, China; Guangdong Provincial Key Laboratory of Insect Developmental Biology and Applied Technology, Guangzhou Key Laboratory of Insect Development Regulation and Application Research, Institute of Insect Science and Technology, School of Life Sciences, South China Normal University, Guangzhou 510631, China; Guangmeiyuan R&D Center, Guangdong Provincial Key Laboratory of Insect Developmental Biology and Applied Technology, South China Normal University, Meizhou 514779, China; Guangdong Provincial Key Laboratory of Insect Developmental Biology and Applied Technology, Guangzhou Key Laboratory of Insect Development Regulation and Application Research, Institute of Insect Science and Technology, School of Life Sciences, South China Normal University, Guangzhou 510631, China; Guangdong Provincial Key Laboratory of Insect Developmental Biology and Applied Technology, Guangzhou Key Laboratory of Insect Development Regulation and Application Research, Institute of Insect Science and Technology, School of Life Sciences, South China Normal University, Guangzhou 510631, China; Guangdong Provincial Key Laboratory of Insect Developmental Biology and Applied Technology, Guangzhou Key Laboratory of Insect Development Regulation and Application Research, Institute of Insect Science and Technology, School of Life Sciences, South China Normal University, Guangzhou 510631, China; Guangdong Provincial Key Laboratory of Insect Developmental Biology and Applied Technology, Guangzhou Key Laboratory of Insect Development Regulation and Application Research, Institute of Insect Science and Technology, School of Life Sciences, South China Normal University, Guangzhou 510631, China; Guangdong Provincial Key Laboratory of Insect Developmental Biology and Applied Technology, Guangzhou Key Laboratory of Insect Development Regulation and Application Research, Institute of Insect Science and Technology, School of Life Sciences, South China Normal University, Guangzhou 510631, China; Department of Entomology, University of Maryland, College Park, MD 20742, USA; Guangdong Provincial Key Laboratory of Insect Developmental Biology and Applied Technology, Guangzhou Key Laboratory of Insect Development Regulation and Application Research, Institute of Insect Science and Technology, School of Life Sciences, South China Normal University, Guangzhou 510631, China; Guangmeiyuan R&D Center, Guangdong Provincial Key Laboratory of Insect Developmental Biology and Applied Technology, South China Normal University, Meizhou 514779, China

**Keywords:** membrane receptor, endoplasmic reticulum, calcium signaling, mitochondrial activity, transcriptional regulation, epigenetic regulation

## Abstract

Inter-organelle communication coordinates cellular homeostasis and function. Juvenile hormone (JH) is produced in the corpora allata (CA) and acts as a gonadotrophic hormone in most insects. Using transcriptomic, biochemical, molecular, and genetic analyses, here we investigated the underlying mechanism of how inter-organelle communication dynamically orchestrates JH biosynthesis and female reproduction in the American cockroach, *Periplaneta americana*. The extracellular stimuli insulin and allatostatin act through their membrane receptors and antagonistically regulate RyR-mediated Ca^2+^ release from the endoplasmic reticulum in CA cells. Ca^2+^-activated CaMKII stimulates energy metabolism in the mitochondria partially via *SLC25A6*, and induces the expression of JH biosynthetic genes *HMGR*, *Jhamt*, and *Cyp15a1* through activating transcription factor CREB, which recruits CBP for histone acetylation in the nucleus. Additionally, mitochondria interact with CREB-CBP through mitonuclear communication to regulate JH biosynthesis. From the perspective of inter-organelle communication, this comprehensive study significantly advanced our understanding of hormone biosynthesis and reproductive biology in insects.

## INTRODUCTION

In the complex realm of cell biology, inter-organelle communication is emerging as a fundamental aspect of life-sustaining processes. Organelles, such as mitochondria, endoplasmic reticulum (ER), nucleus, plasma membrane, Golgi apparatus, peroxisomes, lipid droplets, and lysosomes, represent functional entities that are responsible for specific tasks, and engage in inter-organelle communication within eukaryotic cells. Inter-organelle communication encompasses both indirect and direct interactions between organelles, involving signal transduction, small molecule and ions exchange, and specialized membrane contact sites [1–4]. Elucidating the intricate connectivity of organelle networks is crucial for comprehending organismal homeostasis and function in adaptation to changing physiological states and external stimuli [1,2,5–7]. Hormone biosynthesis in endocrinal cells relies on numerous organelles working in concert. As an example, steroid hormones are produced through the coordinated efforts of mitochondria, ER, and other organelles [5,8]. However, how inter-organelles coordinately regulate hormone biosynthesis and thus reproduction is still poorly understood.

Juvenile hormone (JH), a class of structurally unique sesquiterpenoid hormones in insects, plays crucial roles in regulating metamorphosis, reproduction, embryogenesis, diapause, and caste differentiation [9–16]. Notably, JH acts as a pivotal gonadotrophic hormone facilitating female reproduction in most insect species [17]. The variant JH III predominates in Insecta [9], including cockroaches. JH biosynthesis occurs within the corpora allata (CA), a pair of small endocrine glands connected to the insect brain. JH III biosynthesis involves 13 distinct enzymes, with three playing key roles: hydroxymethylglutaryl-CoA reductase (HMGR) is a key enzyme in the early mevalonate pathway, while juvenile hormone acid methyltransferase (Jhamt) and methyl farnesoate epoxidase (Cyp15a1) catalyze the final two JH-specific steps [9,18–20]. Extensive research has identified many factors that modulate JH biosynthesis across insects and developmental stages, including nutrition-sensitive insulin/IGF signaling (IIS) and target of rapamycin complex1 (TORC1) [17,21,22], Egfr signaling [23], ecdysis triggering hormone (ETH) [24], Dpp signaling [25] and calcium signaling [26–28], which promote JH biosynthesis, whereas neuropeptide allatostatin (AST) [29–31], 20-hydroxyecdysone (20E) [32,33] and microRNAs [17] inhibit JH biosynthesis. Among these signals, extracellular cues (i.e. insulin and AST) act through their membrane receptors to modulate intracellular signal transduction pathways [21,29], such as calcium signaling in which intracellular Ca^2+^ release is mediated by the ryanodine receptor (RyR) and inositol 1,4,5-trisphosphate receptor (IP3R) localized on the ER [34,35]. Many studies have reported that different organelles (ER, mitochondria and peroxisomes) participate in JH biosynthesis [9,36–38]. However, the underlying mechanisms and physiological significance of how inter-organelle communication regulates JH biosynthesis in CA cells have never been extensively investigated.

The American cockroach, *Periplaneta americana*, is an excellent model for studying the regulation of JH biosynthesis, in part due to its relatively large CA size and high levels of JH biosynthesis during female reproduction [21,39]. We have previously documented that IIS-TORC1 dynamically promotes JH biosynthesis and female reproduction during its first gonadotrophic cycle [21,39]*.* In this study, we initially conducted a comprehensive transcriptome analysis of the CA and found that a significant number of genes related to mitochondria and calcium signaling are upregulated during the peak of JH biosynthesis. We then revealed that both mitochondrial energy metabolism and RyR-mediated Ca^2+^ release from the ER are essential for JH biosynthesis and female reproduction. Further, we showed that an intricate inter-organelle communication among the ER, mitochondria, and nucleus in CA cells coordinately controls these processes. Finally, we found that insulin and allatostatin act through their membrane receptors to dynamically modulate RyR-mediated Ca^2+^ release from the ER, linking extracellular cues to the intricate inter-organelle communication. Our findings provide fundamental insights into how inter-organelle communication controls insect hormone biosynthesis and female reproduction, significantly advancing our understanding of insect endocrinology and reproductive biology.

## RESULTS

### Transcriptome analyses reveal potential roles of mitochondria and calcium signaling

**Figure 1.**
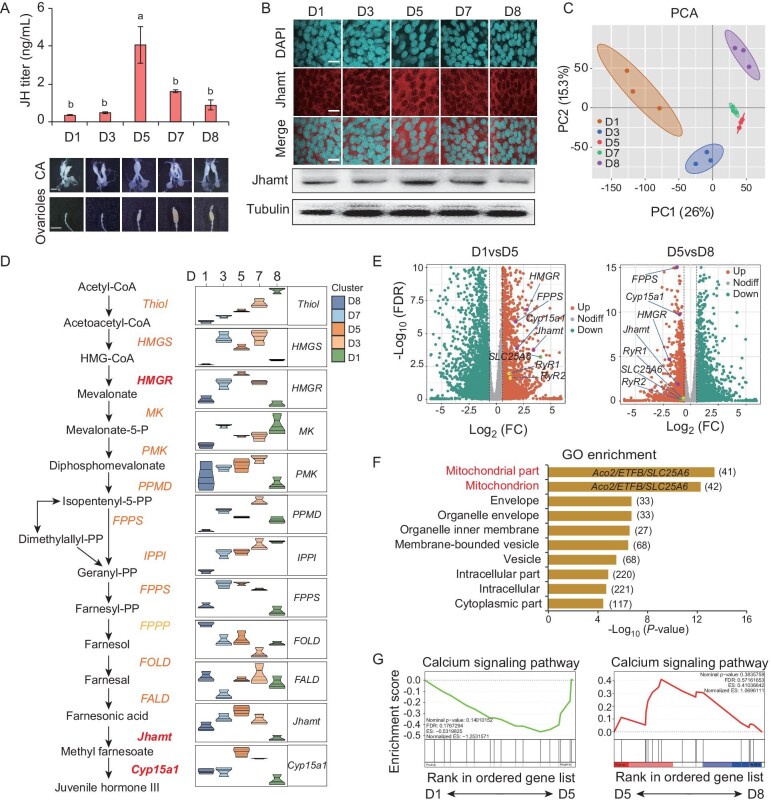
Transcriptome analysis of enriched genes in mitochondria and calcium signaling. (A) Dynamic changes in JH titer, as well as CA and ovaries’ morphology at different time points during the first gonadotrophic cycle. D1 indicates day 1 post adult emergence (PAE), and etc. The representation also applies to the following legend. (B) Immunofluorescence and western blotting analysis of Jhamt during the first gonadotrophic cycle. CA tissues were collected for western blotting. (C) PCA of samples at different time points. (D) Heatmap of genes involved in the JH biosynthetic pathway. (E) Volcano plots of differentially expressed genes between D1 and D5, as well as between D5 and D8. (F) GO enrichment analysis of cellular components associated with upregulated genes on D5 compared to D1 and D8. The horizontal axis represents the significance of differences in enriched gene sets or signaling pathways. The number adjacent to each bar on the chart indicates the total number of genes contained within a specific gene set or pathway. (G) Calcium signaling pathway was upregulated in D5 compared to D1 and D8. Scale bars: 300 μm (A, CA), 2 mm (A, ovary), 20 μm (B). Data are mean ± SEM. *n* = 3 or 4. Different letters indicate statistically significant differences (*P *< 0.05).

To gain a deeper understanding of JH regulation of female reproduction in the American cockroach [21,39], we first quantified JH titer in the hemolymph of female cockroaches during the first gonadotrophic cycle using liquid chromatography with tandem mass spectrometry (LC-MS/MS). We collected hemolymph samples at multiple time points post adult eclosion (PAE), specifically on days 1, 3, 5, 7 and 8 (D1/D3/D5/D7/D8). The LC-MS/MS results showed a significant increase in JH titer during the first half of the gonadotrophic cycle, with a sharp peak on D5, followed by a steady decline until D8 (Fig. 1A). The protein levels of Jhamt exhibited a dynamic fluctuation mirroring the changes in JH titer (Fig. 1B; Fig. S1A and B) and the developmental expression profile of *Kr-h1* [21], a JH primary-response gene that represents JH intracellular signaling [10].

Then, we performed transcriptome analyses of the CA at the above-mentioned time points. Principal component analysis (PCA) clearly showed that samples from different time points were distinct, with minimal variation observed within the D5 and D7 samples (Fig. 1C). A heatmap depicting the developmental expression profiles of enzyme-coding genes in the JH biosynthetic pathway showed that the expression patterns of approximately half of these genes closely mirrored the developmental fluctuations in Jhamt protein levels, JH titer and *Kr-h1* expression (Fig. 1D) [21]. These results suggest that those fluctuating JH biosynthetic genes are largely responsible for JH biosynthesis and thus JH titer in a dynamic manner. In the mevalonate pathway, *HMGR* exhibits the highest expression level, while in JH-specific steps, the expression of *Jhamt* and *Cyp15a1* are prominent, thereby making them significant indicators of JH biosynthesis (Fig. 1D). In addition, the gene coding farnesyl diphosphate synthase (FPPS) is likely to be another key fluctuating JH biosynthetic gene (Fig. 1D).

Since JH levels peak on D5, we conducted comparisons between D1 and D5 as well as between D5 and D8 to identify differentially expressed genes (DEGs) in the CA. The analysis aims to screen for potential regulatory genes and pathways that govern JH biosynthesis. We discovered that 1890 genes were upregulated on D5 compared to D1, and 1521 genes that were upregulated on D5 compared to D8 (fold change >1.4, *P*-value < 0.3). A total of 525 upregulated genes on D5 were identified as being involved in both comparisons, including *HMGR*, *Jhamt* and *Cyp15a1* (Fig. 1E, purple dots, and Fig. S1C). Significantly, mitochondria-associated genes were top-ranked in Gene Ontology (GO) (marked in red) (Fig. 1F). As most of the genes in these two gene sets overlapped, only 42 mitochondria-associated genes were identified in total (Fig. S1D). Gene set enrichment analysis (GSEA) revealed that the 525 overlapping genes were notably enriched in the tricarboxylic acid cycle (TCA cycle), oxidative phosphorylation (OXPHOS) and fatty acid degradation (Fig. S1E), including the 42 mitochondria-associated genes. Notably, GSEA also showed that calcium signaling pathway was prominent, with 17 genes identified (Fig. 1G and Fig. S1F). This gene set included two ryanodine receptor genes, *RyR1* and *RyR2*, which play a pivotal role in maintaining intracellular calcium homeostasis [34,35] (Fig. 1E, yellow dots, and Fig. S1F). Moreover, the gene coding solute carrier family 25 member 6 (SLC25A6), an ADP:ATP antiporter belonging to the mitochondrial carrier family with a calcium-regulated domain [40], was enriched in both the calcium signaling pathway and mitochondria-associated genes (Fig. 1E, green dots, F and Fig. S1D and F). The transcriptomic analysis indicates that mitochondria and calcium signaling may work together to regulate JH biosynthesis, possibly via inter-organelle communication.

### Mitochondrial energy metabolism is indispensable for JH biosynthesis

**Figure 2.**
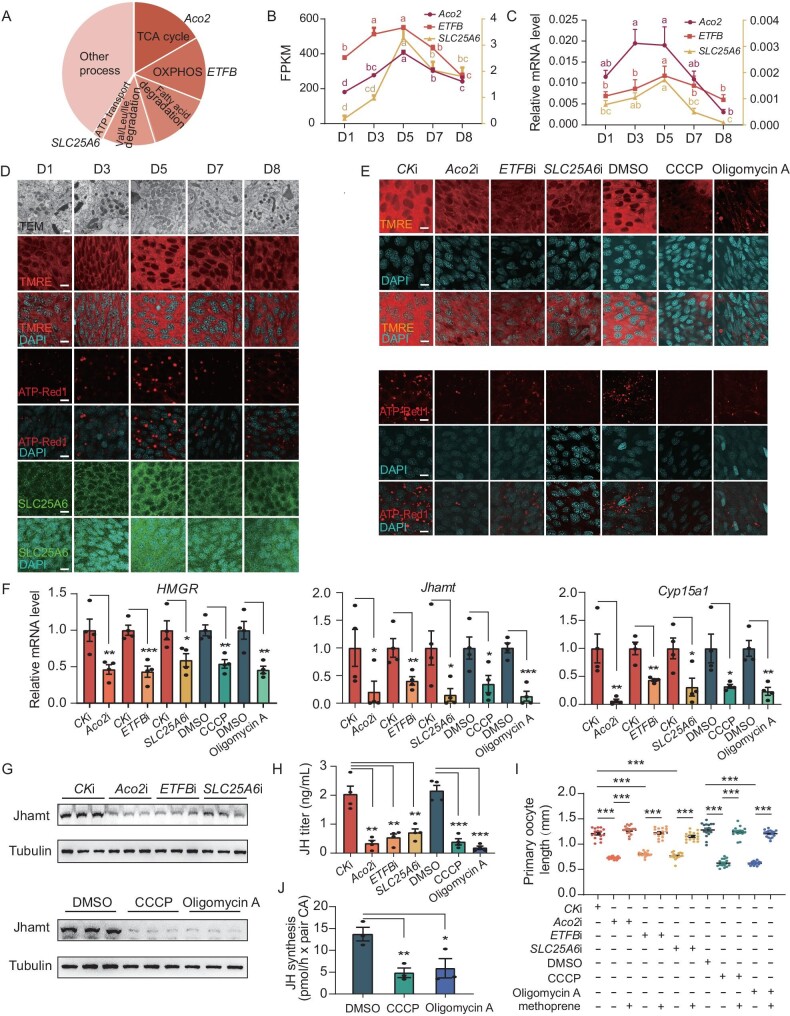
Mitochondrial energy metabolism is indispensable for promoting JH biosynthesis. (A) Classification of the 42 mitochondria-associated genes shown in Fig. 1F. (B) Transcriptome profiles show the expression patterns of *Aco2*, *ETFB* and *SLC25A6* during the first gonadotrophic cycle. (C) Expression patterns of *Aco2*, *ETFB* and *SLC25A6* detected by qPCR. (D) Dynamic changes of mitochondrial morphology and metabolic activity during the first gonadotrophic cycle. Mitochondrial morphology was observed using transmission electron microscopy (TEM). Mitochondrial activity was evaluated using tetramethylrhodamine ethyl ester (TMRE) and ATP Red1. SLC25A6 protein levels were detected using immunofluorescence. (E) Detection of SLC25A6, TMRE and ATP Red1 levels following disruption of mitochondrial activity. Mitochondrial activity was blocked by knockdown of *Aco2*, *ETFB* and *SLC25A6,* as well as treatment with the inhibitors CCCP and oligomycin A. Fluorescence was quantified using Photoshop and normalized to control. A 92-bp noncoding sequence from the pSTBlue-1 vector was used to generate CK dsRNA as a control, suitable for use in the following experiments. (F) Detection of *HMGR*, *Jhamt* and *Cyp15a1* mRNA level following disruption of mitochondrial activity. *n* = 4. (G) Detection of Jhamt protein levels by western blotting following disruption of mitochondrial activity using CA tissues. *n* = 3. (H) Determination of JH titer by LC-MS/MS following disruption of mitochondrial activity. *n* = 4. (I) Measurements of primary oocyte length following the reduction of mitochondrial activity and subsequent rescue with methoprene (JH mimic). *n* = 16. (J) Determination of JH biosynthesis *in vitro* following the suppression of mitochondrial activity using CCCP and oligomycin A. *n* = 4. Scale bars: 500 nm (D, TEM), 20 μm (D, SLC25A6, TMRE and ATP-Red 1). Data are mean ± SEM. Different letters indicate statistically significant differences (*P *< 0.05). *P < 0.05, **P < 0.01, ***P < 0.001, compared to the negative control (CK RNAi or DMSO). *n* = 3 or 4.

Mitochondria are dynamic and living organelles that play crucial roles in energy transformation, biosynthesis, and signaling that enhance organismal adaptation [7]. The 42 enriched mitochondria-associated genes (Fig. S1D) are mainly involved in mitochondrial energy metabolism, encompassing key processes such as TCA cycle, OXPHOS and fatty acid degradation (Fig. 2A). We strategically selected one of the most highly expressed genes from each process to investigate its function in regulating JH biosynthesis, with *Aconitase 2* (*Aco2*) in the TCA cycle and *electron transfer flavoprotein beta subunit* (*ETFB*) in OXPHOS (Fig. 2A and S1D). Additionally, *SLC25A6* was also highlighted, as it was enriched in both mitochondrion-related gene sets and calcium signaling pathway (Fig. S1D and F). As confirmed by quantitative real-time PCR (qPCR), the developmental expression patterns of these three mitochondria-associated genes mirrored the changes of JH biosynthesis (Fig. 2B–C).

Notably, we observed that the number of mitochondria in CA cells exhibited dynamic changes, mirroring the fluctuations in JH production throughout the first gonadotrophic cycle (Fig. 2D and Fig. S2A). Using tetramethylrhodamine ethyl ester (TMRE) and ATP-Red 1 staining to assess mitochondrial membrane potential and ATP production, respectively, we observed that mitochondrial energy metabolism exhibited concurrent fluctuations with JH biosynthesis (Fig. 2D and Fig. S2B). Moreover, immunofluorescence revealed a similar developmental profile of the SLC25A6 protein levels (Fig. 2D and Fig. S2B). Correlation analysis revealed a significant positive correlation between these mitochondrial-related indicators and JH biosynthesis (Fig. S2C). These findings strongly support the potential role for mitochondrial energy metabolism in regulating JH biosynthesis.

To verify this hypothesis, we performed RNA interference (RNAi) experiments to deplete the expression of *Aco2*, *ETFB* and *SLC25A6*. The knockdown of these genes successfully suppressed their expression (Fig. S2D–E). Importantly, the knockdown not only suppressed mitochondrial activity in CA cells (Fig. 2E and Fig. S2F), but also decreased JH biosynthetic gene expression and JH titer (Fig. 2F–H and Fig. S2G–H) and female reproduction (Fig. 2I and Fig. S2J). Similar effects occurred upon treatment with OXPHOS inhibitors CCCP and oligomycin A (Fig. 2E–I and Fig. S2F–J). Strikingly, simultaneous treatment with the JH analog methoprene restored the impaired ovarian development caused by knockdown of *Aco2*, *ETFB*, and *SLC25A6* and treatment with CCCP and oligomycin A (Fig. 2I and Fig. S2J). Moreover, when cultured *in vitro*, the CA exhibited reduced mitochondrial activity (Fig. S2I–I’) and diminished JH biosynthesis (Fig. 2J) in response to CCCP and oligomycin A. These findings demonstrate the indispensable role of mitochondrial energy metabolism in promoting JH biosynthesis within CA cells.

### RyR-mediated Ca^2+^ release from the ER stimulates JH biosynthesis via CaMKII

**Figure 3.**
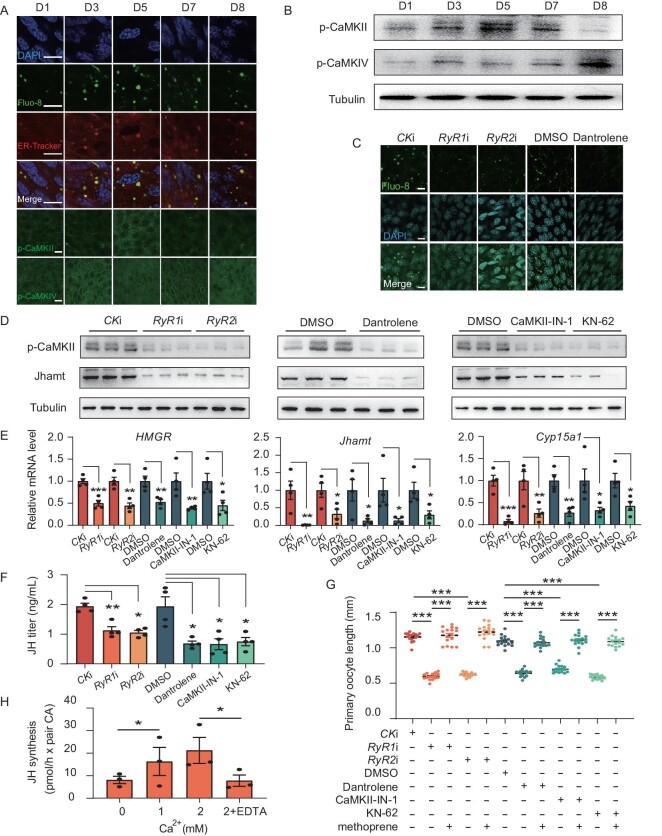
RyR-mediated Ca^2+^ release from the ER and CaMKII activation facilitates JH biosynthesis. (A) Detection of Ca^2+^, phosphorylated CaMKII (p-CaMKII) and phosphorylated CaMKIV (p-CaMKIV) developmental patterns, as well as subcellular location of Ca^2+^ in CA cells. Ca^2+^ was stained with Fluo-8 dye. (B) Patterns of p-CaMKII and p-CaMKIV. CA tissues were collected for western blotting. (C) Immunofluorescence analysis of Fluo-8 following knockdown of *RyR1* and *RyR2*, as well as treatment with the inhibitor Dantrolene. (D) Detection of p-CaMKII and Jhamt by western blotting following disruption of calcium signaling using CA tissues. (E) Detection of *HMGR*, *Jhamt* and *Cyp15a1* mRNA level following disruption of calcium signaling. (F) Determination of JH titer by LC-MS/MS following disruption of calcium signaling. (G) Measurements of primary oocyte length following the reduction of calcium signaling and subsequent rescue with methoprene (JH mimic). (H) Determination of JH biosynthesis *in vitro* following treatment with Ca^2+^ and the chelating agent EDTA. Scale bars: 20 μm. Data are mean ± SEM. *P < 0.05, **P < 0.01, ***P < 0.001, compared to the negative control (CK RNAi or DMSO).

Our transcriptomic analysis suggests that *RyR1* and *RyR2*, rather than *IP3R*, mediate Ca^2+^ release from the ER in regulating JH biosynthesis (Fig. 1G). RyRs are intracellular Ca^2+^ channels located in the ER that are critical for mediating Ca^2+^ release, and are integral to processes such as muscle contraction and neuronal function. We also observed that the developmental profiles of *RyR1* and *RyR2* expression exhibited a concordant fluctuation with that of JH biosynthesis, while *IP3R* did not (Fig. S3A–B). Notably, significant co-localization of Ca^2+^ and the ER was observed using Fluo-8 staining and ER-Tracker, respectively (Fig. 3A). Furthermore, intracellular Ca^2+^ levels fluctuated with a peak observed at D5 PAE, in accordance with the JH biosynthesis pattern (Fig. 3A and Fig. S3B–C). To gain insight into calcium signaling in CA cells, we measured the activities of Ca^2+^/calmodulin-dependent protein kinases (CaMKs), i.e. CaMKII and CaMKIV, which can be activated by the intracellular Ca^2+^ [41,42]. The immunohistochemistry and western blotting results show that the levels of phosphorylated CaMKII (p-CaMKII), but not phosphorylated CaMKIV (p-CaMKIV) (Fig. 3A–B and Fig. S3B–D), exhibited a strong correlation between Ca^2+^ signaling and JH biosynthesis during the first gonadotropic cycle.

As expected, RNAi knockdown of *RyR1* and *RyR2* resulted in a significant reduction in gene expression, while RyR inhibitor Dantrolene treatment had similar effects (Fig. S3E), indicating a positive regulatory loop in Ca^2+^ signaling and *RyR1* and *RyR2* expression. The knockdown significantly reduced intracellular Ca^2+^ levels (Fig. 3C and Fig. S3F) and p-CaMKII levels (Fig. 3D and Fig. S3G–H). It is noteworthy that the knockdown repressed the expression of JH biosynthetic genes *HMGR*, *Jhamt* and *Cyp15a1* (Fig. 3E), the protein levels of Jhamt (Fig. 3D and Fig. S3G, I), JH titer (Fig. 3F), as well as ovary development (Fig. 3G and Fig. S3J). Treating with CaMKII inhibitors, CaMKII-IN-1 and KN-62, yielded results that were identical to those observed in the *RyRs* knockdown (Fig. 3D–G and Fig. S3G–J). Furthermore, methoprene injection rescued the impaired reproduction caused by calcium signaling suppression (Fig. 3G and Fig. S3J). Finally, an *in vitro* experiment confirmed that Ca^2+^ induced JH biosynthesis, and this induction was able to be suppressed by the Ca^2+^ chelator EDTA (Fig. 3H). In conclusion, RyR-mediated Ca^2+^ release from the ER stimulates JH biosynthesis via CaMKII.

### Ca^2+^ signaling-activated CREB-CBP induces JH biosynthetic gene expression in the nucleus

**Figure 4.**
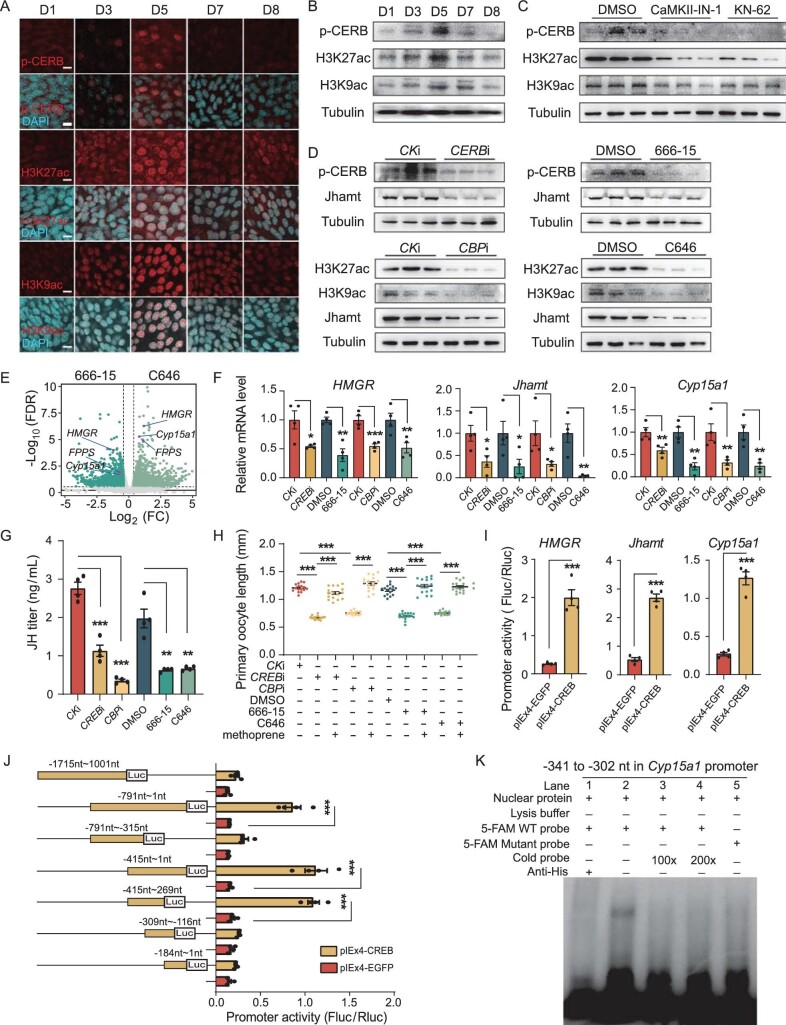
Nucleus-localized CREB-CBP induces the expression of JH biosynthetic genes. (A) Dynamic changes of phosphorylated CREB (p-CREB) and acetylation of H3K27/H3K9 (H3K27ac/H3K9ac) in CA cells during the first gonadotrophic cycle. p-CREB, H3K27ac and H3K9ac were mainly localized in nuclei at all the stages but differed in fluorescence intensity. (B) Detection of the developmental profiles of p-CREB, H3K27ac and H3K9ac by western blotting using CA tissues. (C) Detection of p-CREB, H3K27ac, and H3K9ac following treatment with CaMKII inhibitors CaMKII-IN-1 and KN-62. CA tissues were collected for western blotting. (D) Detection of p-CREB and Jhamt by western blotting using CA tissues following knockdown of *CREB* and treatment with inhibitor 666–15, as well as H3K27ac, H3K9ac and Jhamt following knockdown of *CBP* and treatment with inhibitor C646. (E) Volcano plots of downregulated genes following treatment with 666–15 and C646. (F) Expression of *HMGR*, *Jhamt* and *Cyp15a* following *CREB-CBP* disruption. (G) Determination of JH titer by LC-MS/MS following *CREB-CBP* disruption. (H) Measurements of primary oocyte length following *CREB-CBP* disruption and subsequent rescue with methoprene (JH mimic). (I) Relative luciferase activity of *HMGR* (−2298 nt ∼ −1 nt), *Jhamt* (−2050 nt ∼ −1nt) and *Cyp15a1* (−1715 nt ∼ −1 nt) promoter transfection with pIEx4-CREB. (J) Relative luciferase activity of different regions of the *Cyp15a1* promoter. The orange lines represent the *Cyp15a1* promoter fragments that cloned into the pGL3 vector, and the black lines represent the *Cyp15a1* promoter excised fragment. (K) EMSA analysis of the binding of nuclear proteins CREB extracted from KC cells using a region from −341 to −302 nt in the *Cyp15a1* promoter and the mutated probes or His antibody. Scale bars: 20 μm. *n* = 4. Data are mean ± SEM. *P < 0.05, **P < 0.01, ***P < 0.001, compared to the negative control (CK RNAi or DMSO).

It is well established that Ca^2+^ signaling stimulates gene transcription by activating a variety of transcription factors (TFs). The TF cAMP response element binding protein (CREB) is one of the best-known effectors of calcium signaling [43,44]. When CREB is activated through phosphorylation at Ser-133 by calcium signaling, its phosphorylated form (p-CREB) binds to cAMP response elements (CREs) on promoter regions of the target genes, resulting in the induction of gene expression [43,44]. Meanwhile, p-CREB recruits its coactivator CREB-binding protein (CBP, an epigenetic modifier that modifies chromatin accessibility), which acts as a histone acetyltransferase on histone H3 lysine 27 and 9 (H3K27 and H3K9) to promote CREB-dependent gene expression [45,46]. Thus, we measured p-CREB and CBP-activated H3K27ac/H3K9ac levels in CA cells during the first gonadotrophic cycle, showing that these markers were nuclear-localized and exhibited developmental patterns closely resembling those observed in calcium signaling and JH biosynthesis (Fig. 4A–B and Fig. S4A–C). Then, we established that Ca^2+^ signaling was required for CREB-CBP activation within CA cells (Fig. 4C and Fig. S5).

Subsequently, we examined whether the Ca^2+^-activated CREB-CBP induces JH biosynthesis. We utilized RNAi to knockdown *CREB* and *CBP*, and we also applied the CREB inhibitor 666–15 and the CBP inhibitor C646. These interventions significantly decreased p-CREB and CBP-activated H3K27ac/H3K9ac levels in CA cells, respectively (Fig. 4D and Fig. S4D–F). Then, we conducted a transcriptomic analysis on CA tissues following treatment with the CREB inhibitor 666–15 and the CBP inhibitor C646. PCA showed that the samples of inhibitor-treated groups were distinct from those of the controls (Fig. S6A–B). A Venn diagram analysis identified 188 downregulated genes common to both 666–15 and C646 treatment (Fig. S6C), including the JH biosynthetic genes *HMGR*, *FPPS* and *Cyp15a1* (Fig. 4E, purple dots). Moreover, GO analysis suggested that CREB and CBP regulated many mitochondria-associated genes in CA cells (Fig. S6D–F), revealing a possible mitonuclear communication. Importantly, CREB-CBP suppression led to a decrease in JH biosynthesis (Fig. 4D–G and Fig. S4D–F) and female reproduction (Fig. 4H and Fig. S4G). Furthermore, treatment with methoprene effectively rescued the ovarian impairment induced by CREB-CBP disruption (Fig. 4H and Fig. S4G).

We hypothesized that nucleus-localized CREB may directly target cAMP response element (CRE, which are typically palindromic or half-site sequences [47]) sites in the promoters of JH biosynthetic genes for transcriptional regulation. To test this, we expressed His-tagged cockroach *CREB* in *Drosophila* KC cells (Fig. S4H). A dual-luciferase reporter assay revealed a significant increase in promoter-driven expression for *HMGR* (−2298∼ −1 nt), *Jhamt* (−2050∼ −1 nt) and *Cyp15a1* (−1715∼ −1 nt) in the presence of cockroach CREB (Fig. 4I). Subsequently, we focused on *Cyp15a1*, the biosynthetic gene in the final step of JH biosynthesis, to investigate the potential binding sites of CREs. We generated truncated 5’-flanking regions of the *Cyp15a1* promoter and identified a critical region from −415 to −269 nt that significantly enhanced luciferase activity (Fig. 4J). Besides, we found a CRE half-site–like sequence, TGAGGTCA, located at positions −323 to −316 nt within the *Cyp15a1* promoter. To validate this interaction, we created fluorescence-labeled and cold DNA probes encompassing the sequence from −341 to −302 nt. An electrophoretic mobility shift assay (EMSA) confirmed the specific binding between the His-CREB-containing nuclear protein extract and the fluorescence-labelled probe (Fig. 4K, lane 2). This binding was competitively inhibited by the cold probes (Fig. 4K, lanes 3 and 4) but not by the mutated probe lacking the TGAGGTCA sequence (Fig. 4K, lane 5). Notably, the band's intensity was abolished by preincubation with a His antibody, indicating the specificity of the interaction (Fig. 4K, lane 1). Collectively, our findings demonstrate that Ca^2+^ signaling-activated CREB-CBP induces JH biosynthetic gene expression in the nucleus, highlighting the critical interplay between the ER and the nucleus in modulating JH biosynthesis within CA cells.

### Inter-organelle communication among the ER, mitochondria and nucleus coordinates JH biosynthesis

**Figure 5.**
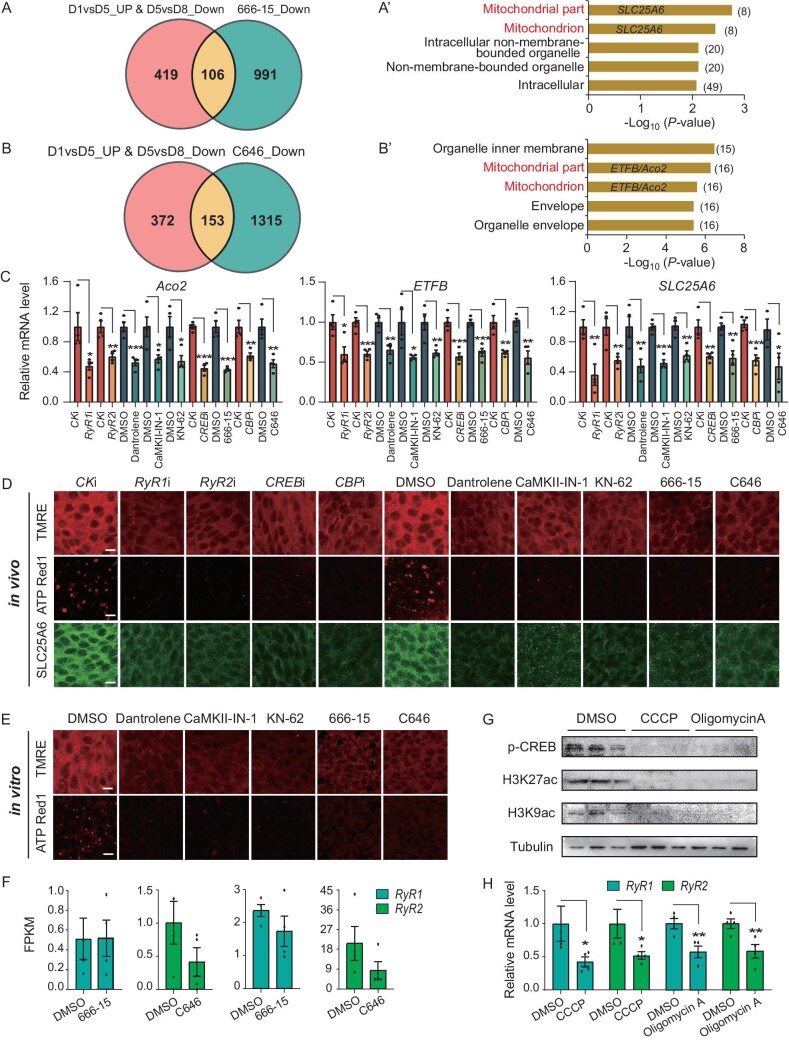
The crosstalk between mitochondrial activity and CREB-CBP transcriptional activity. (A) Venn diagram analysis of D5-upregulated genes and 666–15 inhibited genes. (A’) GO analysis of 106 overlapping genes in (A). Mitochondria-associated gene sets are marked in red. (B) Venn diagram analysis of D5-upregulated genes and C646 inhibited genes. (B’) GO analysis of 153 overlapping genes in (B). Mitochondria-associated gene sets are highlighted in red. The horizontal axis represents the significance of differences in enriched gene sets or signaling pathways. In (A’) and (B’), the number adjacent to each bar on the chart indicates the total number of genes contained within a specific gene set or pathway. (C) Expression of *Aco2*, *ETFB* and *SLC25A6* following disruption of calcium signaling and *CREB-CBP*. (D) Detection of TMRE, ATP-Red 1 and SLC25A6 following disruption of calcium signaling and *CREB-CBP*. (E) Detection of TMRE and ATP-Red 1 in CA tissues cultured *in vitro* following treatment with Dantrolene, CaMKII-IN-1, KN-62, 666–15 and C646. (F) Expression of *RyR1* and *RyR2* following disruption of *CREB* and *CBP* with 666–15 and C646, respectively. (G) Detection of p-CREB, H3K27ac and H3K9ac by western blotting using CA tissues following disruption of mitochondrial activity. (H) Expression of *RyR1* and *RyR2* following disruption of mitochondrial activity with CCCP and oligomycin A. *n* = 3 or 4. Scale bars: 20 μm. Data are mean ± SEM. *P < 0.05, **P < 0.01, ***P < 0.001, compared to the negative control (CK RNAi or DMSO).

The above findings not only demonstrate the roles of ER-nucleus interaction in the regulation of JH biosynthesis, but also suggest the potential mitonuclear communication and ER-mitochondria interaction. We further profiled the transcriptome data to explore the potential inter-organelle communications in this process. A Venn analysis identified 106 overlapping genes between CREB inhibitor-downregulated genes and D5-upregulated genes. The mitochondria-associated genes were ranked highest using GO analysis, with 8 mitochondria-associated genes being identified, including *SLC25A6* (Fig. 5A–A’ and Table S1). Similarly, 153 overlapping genes were found between CBP inhibitor-downregulated genes and D5-upregulated genes, with 16 of these being mitochondria-associated genes, such as *Aco2* and *ETFB* (Fig. 5B–B’ and Table S2). KEGG pathway analysis of these genes indicated an enrichment of the mitochondria-associated genes involved in OXPHOS (Fig. S7A–B), showing that CREB-CBP modulates mitochondrial activity through mitonuclear communication.

The depletion of calcium signaling and CREB-CBP transcriptional activity further confirmed their stimulatory roles in mitochondrial energy metabolism. This is evidenced by a reduction in the expression of *Aco2*, *ETFB* and *SLC25A6* (Fig. 5C), as well as mitochondrial membrane potential and ATP levels (Fig. 5D and Fig. S7C). An *in vitro* culture experiment also corroborated these findings (Fig. 5E and Fig. S7D). Nevertheless, they showed little or insignificant inhibitory effects on *RyR* gene expression (Fig. 5F). Conversely, the inhibition of mitochondrial activity led to a reduction in the levels of p-CREB and CBP-activated H3K27ac/H3K9ac (Fig. 5G and Fig. S7E–F); moreover, it significantly decreased the expression of both *RyR1* and *RyR2* (Fig. 5H), further emphasizing the crucial interplay between mitochondrial function and *RyR* gene regulation and thus ER-mitochondria interaction. The composite data reveal a positive regulatory loop involving calcium signaling, mitochondrial energy metabolism and CREB-CBP, indicating important roles of inter-organelle communication among the ER, mitochondria and nucleus that regulates JH biosynthesis in CA cells.

### Membrane receptors transduce dynamic extracellular cues to calcium signaling for regulating JH biosynthesis

**Figure 6.**
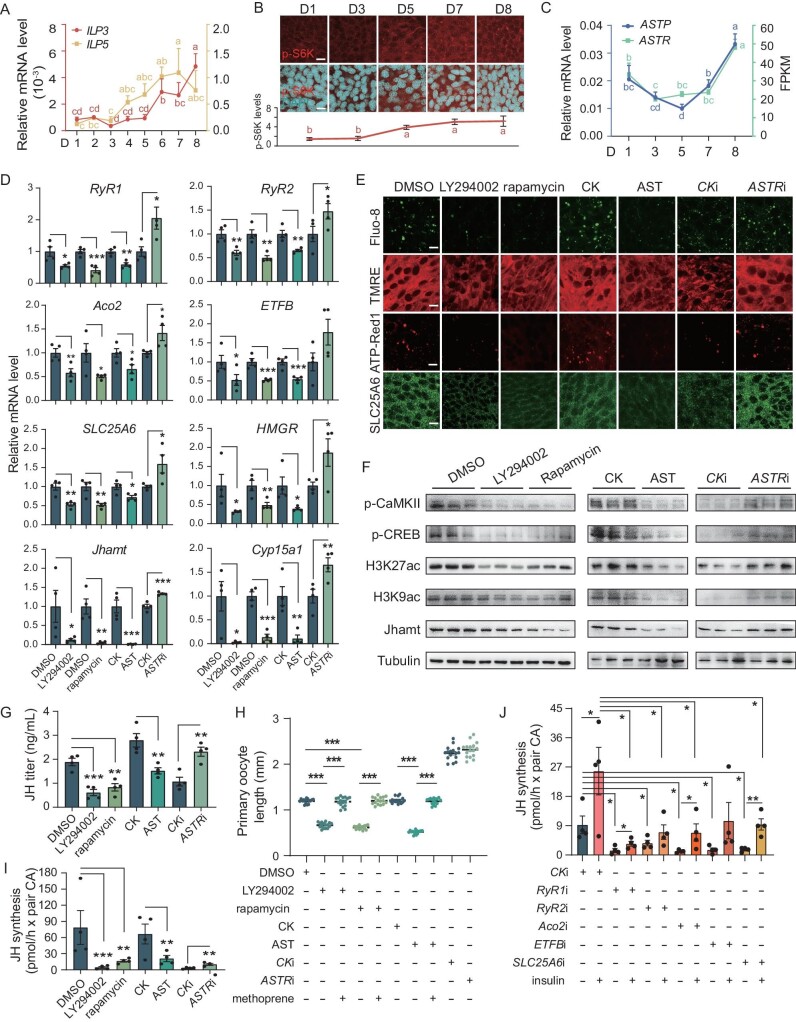
Insulin and AST antagonistically regulate Ca^2+^ release from ER for dynamic orchestration of JH biosynthesis. (A) Expression of *ILP3* and *ILP5* in brains during the first gonadotrophic cycle. *n* = 4. (B) Immunofluorescence analysis of phosphorylated S6K (p-S6K). *n* = 4. (C) Expression of *ASTP* in brains and *ASTR* in CA during the first gonadotrophic cycle. *n* = 4. (D) Expression of *RyR1*, *RyR2*, *Aco2*, *ETFB*, *SLC25A6*, *HMGR*, *Jhamt* and *Cyp15a1* following disruption of IIS-TORC1 with LY294002 and rapamycin, activation of AST signal with AST and inhibition with *ASTR* RNAi. CK serves as the control for AST treatment and is referenced in the subsequent legends. *n* = 4. (E) Fluorescence analysis of Fluo-8, SLC25A6, TMRE and ATP Red1 levels following disruption of IIS-TORC1, as well as AST activation and *ASTR* inhibition. *n* = 4. (F) Detection of p-CaMKII, p-CREB, H3K27ac, H3K9ac and Jhamt by western blotting using CA tissues following disruption of IIS-TORC1, as well as AST activation and *ASTR* inhibition. *n* = 3. (G) Determination of JH titer by LC-MS/MS following disruption of IIS-TORC1, as well as AST activation and *ASTR* inhibition. *n* = 4. (H) Measurement of primary oocyte length following disruption of IIS-TORC1, and activation of AST, followed by rescue with methoprene (JH mimic). *n* = 16. (I) Determination of JH biosynthesis *in vitro* following disruption of IIS-TORC1, as well as AST activation and *ASTR* inhibition. *n* = 4. (J) Determination of JH biosynthesis *in vitro* following disruption of calcium signaling and mitochondrial activity, and subsequent rescue with insulin. *n* = 4. Data are mean ± SEM. Different letters indicate statistically significant differences (*P *< 0.05). *P < 0.05, **P < 0.01, ***P < 0.001, compared to the negative control (DMSO, CK or CK RNAi).

Finally, we examined the upstream signals that likely activate calcium signaling and thus JH biosynthesis. Our previous studies show that insulin-like peptides (ILPs) act through the membrane receptor InR, a tyrosine kinase receptor, to activate IIS and TORC1, thereby promoting JH biosynthesis [21]. In contrast, the neuropeptide AST, acting through its G-protein coupled receptor ASTR, inhibits JH biosynthesis in various insects, including the American cockroach [29]. Based on these findings, we hypothesized that the membrane receptor-transduced extracellular cues exert antagonistic effects on the regulation of dynamic JH biosynthesis during the first gonadotropic cycle. Our observations showed that the expression of *ILP3* and *ILP5* in the brain and phosphorylated S6K (p-S6K), a marker of TORC1 activity, in CA cells, increased progressively during the first gonadotropic cycle (Fig. 6A–B). This aligns with the developmental pattern of ovarian maturation [21]. In stark contrast, the expression of *allatostatin precursor* (*ASTP*) and its receptor *ASTR* showed a dramatic decrease from D1 to D5, followed by an increase until D8, presenting an inverse pattern to JH biosynthesis (Fig. 6C). Correlation analysis confirms that IIS-TORC1 signaling positively correlates with JH biosynthesis, while ASTP/ASTR signaling negatively correlates (Fig. S8A).

To delve into the underlying inter-organelle communication, we blocked the IIS-TORC1 pathway with LY294002 and rapamycin, and we also activated and inhibited AST-ASTR signaling with AST and *ASTR* RNAi, respectively. LY294002 and rapamycin significantly reduced IIS-TORC1 activity (Fig. S8B). It is crucial to note that treatment with LY294002, rapamycin, and AST suppressed calcium signaling by decreasing the expression of *RyR1* and *RyR2*, intracellular Ca^2+^ levels and p-CaMKII levels (Fig. 6D–F and Fig. S8C–D). Furthermore, these treatments caused a clear inhibition of p-CREB and CBP-activated H3K27ac/H3K9ac levels, indicating a decrease in the CREB-CBP activity (Fig. 6F and Fig. S8C–D). The treatments also reduced the expression of mitochondria-associated genes *Aco2*, *ETFB* and *SLC25A6*, as well as SLC25A6 levels, mitochondrial membrane potential and ATP levels (Fig. 6D–E and Fig. S8C). Finally, we observed a significant reduction in the expression of the JH biosynthetic genes *HMGR*, *Jhamt* and *Cyp15a1*, Jhamt protein levels, JH titer and ovary development following these treatments (Fig. 6F–H and Fig. S8C–E). Notably, methoprene treatment rescued the ovarian impairment caused by the inhibition of IIS-TORC1 and AST signaling (Fig. 6H and Fig. S6E). By contrast, *ASTR* RNAi reverses the above process (Fig. 6D–H and Fig. S8C–E).

**Figure 7.**
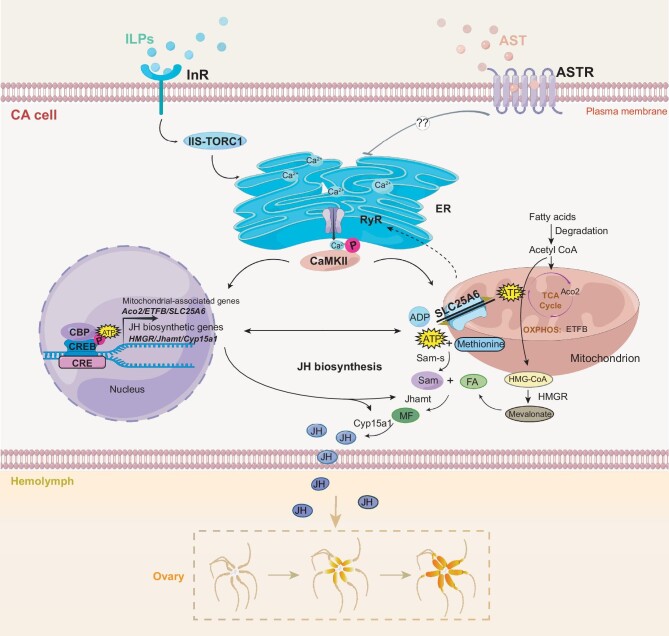
Schematic view of the inter-organelle communication that dynamically orchestrates JH biosynthesis and female reproduction in the American cockroach. During the first gonadotrophic cycle, IIS-TORC1 progressively increases and acts as a stimulator to promote JH biosynthesis. In contrast, AST-ASTR signaling serves as a suppressor, inhibiting JH production in the early and late stages of the cycle. Insulin and AST act through their respective membrane receptors to antagonistically regulate Ca^2+^ release from the ER. This modulates calcium signaling via phosphorylation of CaMKII, which then stimulates the calcium-dependent factors CREB and CBP in the nucleus, inducing the expression of JH biosynthetic genes. In parallel with this nuclear signaling, mitochondrial activity is concurrently stimulated by calcium signaling. Meanwhile, mitonuclear communication acts bidirectionally to regulate JH biosynthesis. In summary, the intricate inter-organelle communication dynamically orchestrates JH biosynthesis and female reproduction in the American cockroach.

Further *in vitro* culture experiments confirmed that insulin induces, while AST inhibits, mitochondrial activity (Fig. S8F) and JH biosynthesis (Fig. 6I). To gain further insight into how IIS-TORC1 is involved in the calcium signaling and mitochondrial metabolism that mediate JH biosynthesis, the CAs from cockroaches after knockdown of *RyR1*, *RyR2*, *Aco2*, *ETFB* and *SLC25A6* were used for *in vitro* culture experiments. These results show that the knockdown significantly reduced JH biosynthesis. Supplementation with insulin significantly induced JH biosynthesis in the control groups and just partially restored JH biosynthesis in the knockdown groups (Fig. 6J). Overall, insulin and allatostatin, acting through their membrane receptors, antagonistically mediate Ca^2+^ release from the ER; calcium signaling then regulates CREB-CBP complex and mitochondrial activity via the intricate inter-organelle communication among the ER, mitochondria and nucleus, orchestrating the dynamic JH biosynthesis in CA cells during the first gonadotrophic cycle (Fig. 7).

## DISCUSSION

### Inter-organelle communication dynamically orchestrates JH biosynthesis

In the present study, we reveal that inter-organelle communication dynamically orchestrates JH biosynthesis and female reproduction in the American cockroach. The ER, serving as a key organelle in calcium signaling transduction mediated by RyR, facilitates communication with the plasma membrane, mitochondria and nucleus to coordinate JH biosynthesis. First, ER-plasma membrane communication is important for the regulation of calcium signaling [48]. Extracellular stimuli insulin and AST act through their respective membrane receptors, InR and ASTR, to antagonistically modulate intracellular calcium signaling. This modulation is the driving force behind the dynamic JH biosynthesis observed during the first gonadotrophic cycle (Fig. 6 and Fig. S8). Second, ER-mitochondria communication facilitates Ca^2+^ release, influencing mitochondrial energy metabolism [49]. Specifically, we demonstrate that RyR-mediated Ca^2+^ release from the ER activates phosphorylation of CaMKII, enhancing mitochondrial activity, thereby increasing JH biosynthesis (Figs 3 and 5). Mitochondrial energy metabolism mediated by *Aco2* and *ETFB*, which are involved in the TCA cycle and OXPHOS, may influence calcium signaling via RyRs (Figs 2 and 5). This interplay suggests a broader role for mitochondrial genes in cellular signaling beyond energy metabolism and ER-mitochondria communication. The activated mitochondria generate acetyl-CoA and ATP, supplying the substrate and energy necessary for JH biosynthesis in the ER (Fig. 2) [29,38]. Interestingly, we have identified a mitochondrial ADP:ATP antiporter, SLC25A6, which contains a calcium-regulatory domain [40], that responds to calcium signaling to promote JH biosynthesis (Figs 2 and 5). This proves that calcium regulation of mitochondrial metabolism-related SLC25A6 is partially involved in ER-mitochondria communication for JH biosynthesis. Conversely, mitochondria exert a feedback regulatory effect on the ER, as evidenced by the modulation of *RyR* gene expression by alterations in mitochondrial metabolism (Fig. 5). Third, ER-nucleus communication enables cells to respond to a range of internal and external cues, regulate gene expression, and maintain essential cellular functions [4]. Our findings demonstrate that calcium signaling triggered by ER-released Ca^2+^ activates nucleus-localized CREB-CBP transcriptional activity (Fig. 4 and Fig. S4). In turn, the CREB-CPB complex potentially regulate the expression of *SLC25A6* and thus Ca^2+^ signaling (Fig. 5), establishing a positive regulatory loop between the ER and the nucleus. In conclusion, the ER plays a central role in regulating JH biosynthesis through extensive calcium signaling that activates the mitochondria and nucleus.

Beyond signaling and molecular exchange, direct physical contacts between organelles are crucial for effective inter-organelle communication. The most well-defined sites of such interactions are the mitochondria-associated ER membranes, which are characterized by junctional complexes, such as the CypD/VDAC1/Grp75/IP3R1 complex [2–4,50,51]. In this study, we noted a significant increase in direct ER-mitochondria contacts at the peak of JH biosynthesis in CA cells (Fig. S9), suggesting enhanced ER-mitochondria communication. However, the detailed molecular composition of these membrane contact sites remains to be elucidated.

Mitochondria and the nucleus engage in mitonuclear communication [52–54] for the regulation of JH biosynthesis. CREB-CBP complex in the nucleus induces the expression of 42 mitochondria-associated genes, which are involved in TCA cycle, OXPHOS, and fatty acid degradation that are important for JH biosynthesis (Figs 2 and 5). Conversely, we demonstrate that mitochondria elicit a retrograde response by producing ATP, potentially through genes involved in the TCA cycle, OXHPOS and ATP transport, including *Aco2*, *ETFB* and *SLC25A6*. This response stimulates CREB-CBP transcriptional activity (Fig. 5), forming a positive regulatory loop. Additionally, mitochondrial metabolites acetyl-CoA and ATP serve as fundamental substrates for JH biosynthesis [38]. Overall, mitonuclear communication, through signaling transduction and ATP transfer, forms a bidirectional loop that stimulates JH biosynthesis. While our study suggests that mitochondria and the nucleus may also communicate through direct contact, future research should aim to identify the protein complexes tethering the membrane sites in CA cells.

### Transcriptional regulation of JH biosynthesis

Our transcriptome analysis showed that the expression of nearly half of the JH biosynthetic genes, such as *HMGS*, *HMGR*, *PPMK*, *FPPS*, *Jhamt* and *Cyp15a1*, exert dynamic expression patterns that peak in the middle of the first gonadotrophic cycle (Fig. 1D). This finding elucidates the fluctuation of JH titer observed during this period. In recent years, the transcriptional regulation of JH biosynthesis mediated by TFs has received considerable attention. Several TFs, such as Vvl, Scr, Mad, Pnt, Seven-up and Ftz-f1, have been shown to transcriptionally regulate JH biosynthesis by targeting the promoter regions of JH biosynthetic genes across various insect species [23,25,55–58]. In this study, we reveal that the fluctuating expression of JH biosynthetic genes is induced by the CREB-CBP complex, as CRE-like elements in the promoter regions of these genes have been identified or predicted (Fig. 4I–L and Table S3). However, genes that do not exhibit fluctuation patterns with JH biosynthesis, such as *MK* and *FALD*, did not alter their expression in response to *CREB-CBP* disruption, nor were they affected by upstream (calcium signaling) and downstream (mitochondrial metabolism) signals (Fig. S10A). Additionally, the expression patterns of *Sam-s*, a gene encoding methionine adenosyltransferase contributing to the synthesis of methyl farnesoate [59], also do not fluctuate with JH biosynthesis (Fig. S10B). This suggests that these unfluctuating genes may not be involved in the transcriptional regulation of JH biosynthesis.

It is important to note that the seminal research by Sutherland and Feyereisen [30] suggested that in the cockroach *Diploptera punctata*, the neuropeptide AST probably affects the transport of citrate across the mitochondrial membrane and/or its cleavage to produce cytoplasmic acetyl-CoA, thereby regulating JH biosynthesis. Given that the observation was based on *in vitro* experiments, we infer that this represents a rapid response. Our *in vitro* experiments, which involved a 4-hour incubation, corroborated this finding (Fig. 6I). Furthermore, in this study, we have elucidated that AST exerts a long-term effect when administered *in vivo* for 48 hours. It modulates the expression of mitochondria-associated genes and JH biosynthetic genes through the Ca^2+^ signaling-activated CREB-CBP complex (Figs 2–6 and Table S3).

In summary, our findings elucidate the intricate inter-organelle communication network among the ER, plasma membrane, mitochondria and nucleus to regulate JH biosynthesis (Fig. 7). This research paves the way for further exploration of the molecular and cellular mechanisms by which inter-organelle communication orchestrates hormone biosynthesis and reproduction in insects.

## MATERIALS AND METHODS

Further information on materials used to conduct the research and methodological details used in the analyses are available in the Supplementary  File.

## Supplementary Material

nwaf022_Supplementary_File
