# Supplementary material for: Inter-organelle communication dynamically orchestrates juvenile hormone biosynthesis and female reproduction
Source: Natl Sci Rev. 2025 Jan 23;12(3):nwaf022. doi: 10.1093/nsr/nwaf022 (PMC11879407; doi:10.1093/nsr/nwaf022)
Supplement: nwaf022_Supplementary_File [file nwaf022_supplementary_file.docx]

**Supporting data for**

Inter-organelle communication dynamically orchestrates juvenile hormone biosynthesis and female reproduction

Shiming Zhu^1, 2, †^, Fangfang Liu^1, 2, †^, Xiaoyi Chen^1, †^, Sishi Xia^1^, Yingting Wu^1^, Wei Tang^1^, Chonghua Ren^1^, Jian Wang^3^, Sheng Li^1, 2, *^

*^1^Guangdong Provincial Key Laboratory of Insect Developmental Biology and Applied Technology, Guangzhou Key Laboratory of Insect Development Regulation and Application Research, Institute of Insect Science and Technology, School of Life Sciences, South China Normal University, Guangzhou 510631, China.*

*^2^Guangmeiyuan R&D Center, Guangdong Provincial Key Laboratory of Insect Developmental Biology and Applied Technology, South China Normal University, Meizhou 514779, China*

*^3^Department of Entomology, University of Maryland, College Park, MD 20742*

*Corresponding authors. Email: lisheng@scnu.edu.cn

^†^Equally contributed to this work.

**This PDF file includes:**

Figures S1 to S10

Tables S1 to S6

Materials and methods text

References


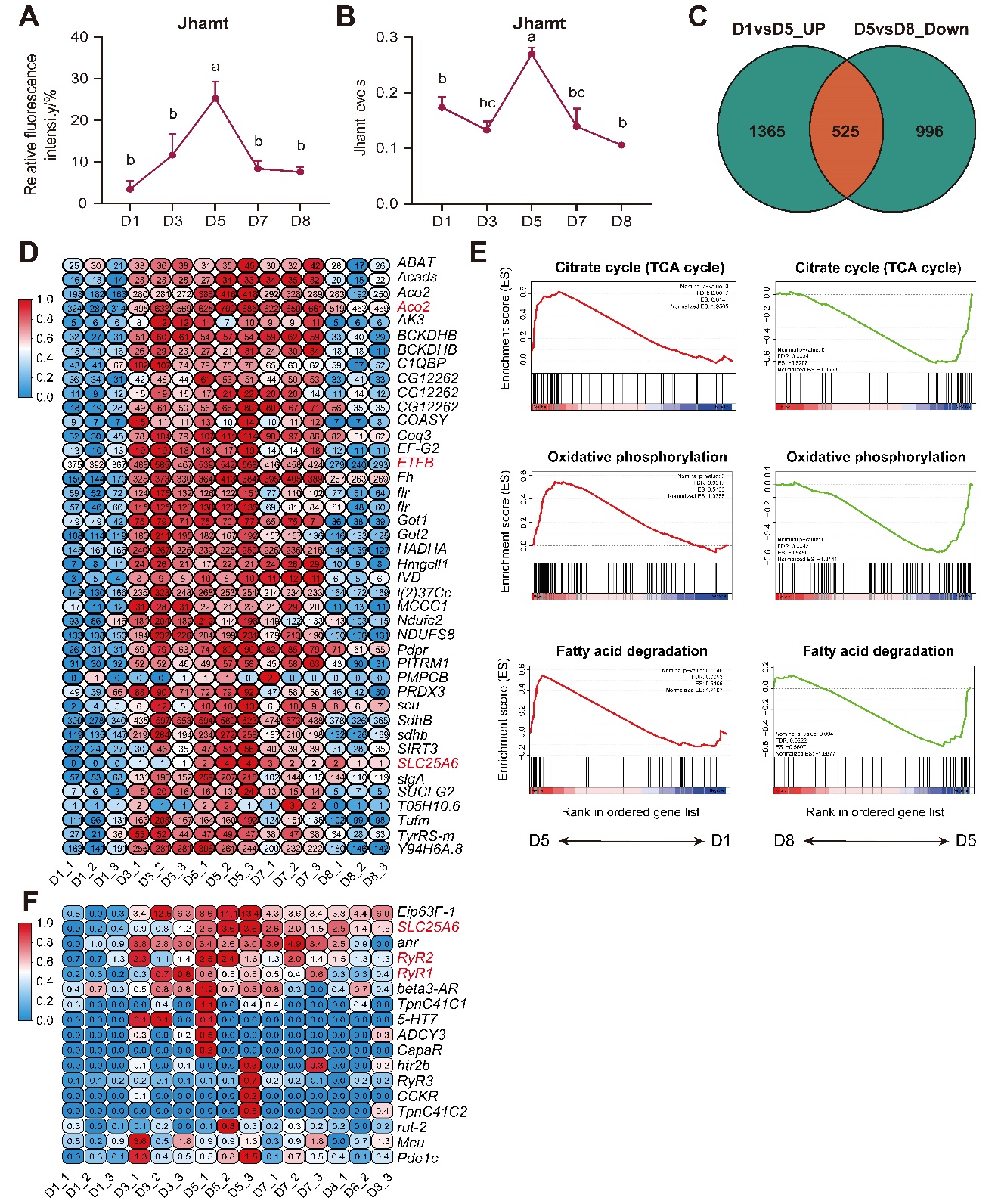


**Figure S1. Statistics of Jhamt protein levels and analysis of genes related to mitochondria and calcium signaling. Related to Figure 1.** (**A**) Quantification of Jhamt levels from Fig 1A. Immunofluorescence was quantified using Photoshop. n=4. (**B**) Quantification of Jhamt levels from Fig. 1B. Western blotting bands were quantified using ImageJ software and normalized to Tubulin levels. n=3. (**C**) Overlap of genes upregulated on D5 compared to D1 and D8. Fold change>1.4, *P*-value<0.3. n=3. (**D**) Heatmap of 42 mitochondria-associated genes enriched from Fig. 1F. n=3. (**E**) Gene set enrichment analysis on pathways that were upregulated on D5 compared to D1 and D8. Three key pathways associated with mitochondrial energy metabolism—the TCA cycle, OXPHOS, and fatty acid degradation—were found to be enriched in both comparisons. (**F**) Heatmap of 17 genes associated with the calcium signaling pathway from Fig 1G. n=3. Data are mean ± SEM. Different letters indicate statistically significant differences (*P*<0.05).


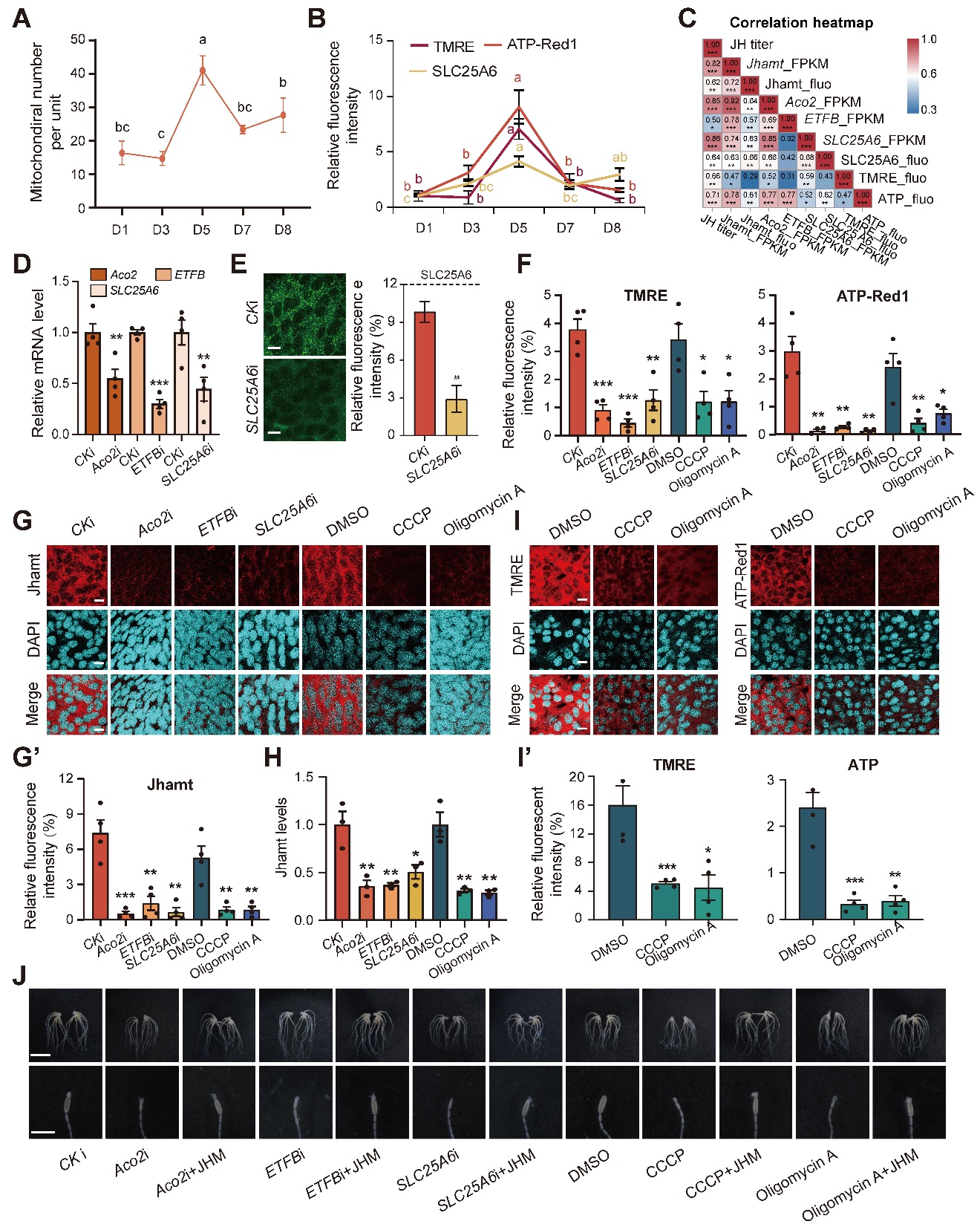


**Figure S2. Mitochondrial activity correlates with JH biosynthesis and female reproduction.** **Related to Figure 2.** (**A**) Quantitative analysis of mitochondrial numbers was ascertained from the TEM images depicted in Fig. 2D. n=3. (**B**) Quantification of TMRE, ATP Red1, and SLC25A6 obtained from the images depicted in Fig. 2D. Fluorescence was quantified using Photoshop and normalized to their levels in D1 PAE, respectively. n=4. (**C**) Correlation analysis indicators reflecting mitochondrial activity in relation to those of reflecting JH biosynthesis. Spearman correlation analysis was conducted utilizing the online platform Omicshare. The numbers in the matrix denote the correlation coefficients. (**D**) RNAi knockdown efficiency of *Aco2*, *ETFB* and *SLC25A6*. n=4. (**E**) Immunofluorescence and quantification of SLC25A6 following *SLC25A6* knockdown. Fluorescence was quantified using Photoshop and normalized to control. n=4. (**F**) Quantification of TMRE and ATP Red1 levels obtained from the images depicted in Fig. 2E. Fluorescence was quantified using Photoshop and normalized to control. n=4. (**G and G’**) Immunofluorescence and quantification of Jhamt levels following knockdown of *Aco2*, *ETFB* and *SLC25A6,* as well as treatments with CCCP and oligomycin A. Fluorescence was quantified using Photoshop and normalized to control. n=4. (**H**) Quantification of Jhamt levels from Fig. 2G. Western blotting bands were quantified using ImageJ software and normalized to Tubulin levels. n=3. (**I and I’**) Fluorescence and quantification of TMRE and ATP Red1 levels following in vitro treatments with inhibitors CCCP and oligomycin A. (**J**) Ovary morphology following depletion of mitochondrial metabolic activity and rescue with methoprene (JH mimic). Fluorescence was quantified using Photoshop and normalized to control. n=4. Scale bars: 20 μm (G, I), 5 mm (J, upper); 2 mm (J, lower). Data are mean ± SEM. Different letters indicate statistically significant differences (*P*<0.05). **P*<0.05, ***P*<0.01, ****P*<0.001.

**
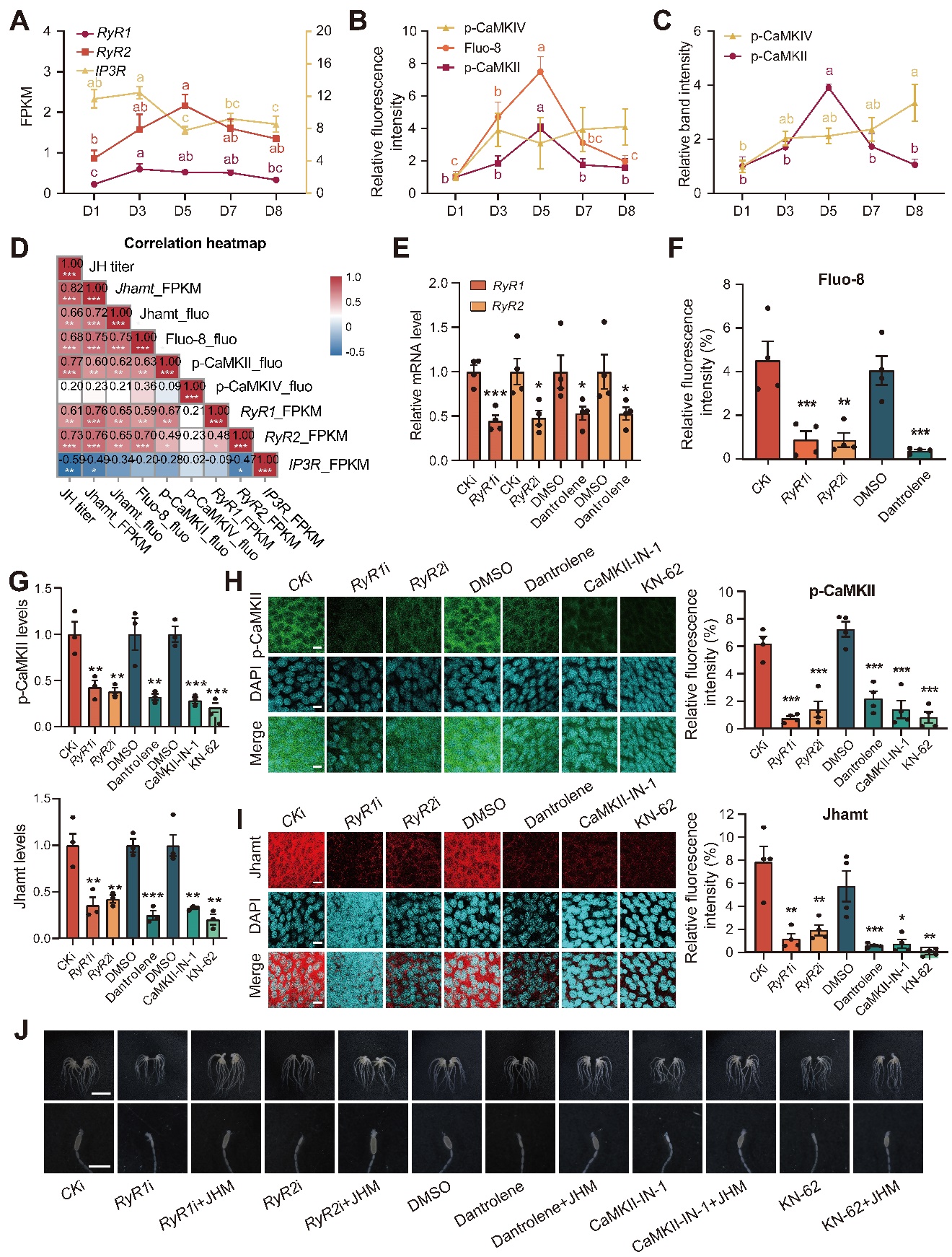
**

**Figure S3. Calcium signaling correlates with JH biosynthesis and female reproduction. Related to Figure 3.** (**A**) Expression patterns of *RyR1*, *RyR2*, and *IP3R* obtained from CA transcriptome analysis in Fig. 1. n=3. (**B**) Correlation analysis indicators reflecting calcium signaling pathway in relation to those of reflecting JH biosynthesis. Spearman correlation analysis conducted utilizing the online platform Omicshare. (**C**) Quantification of Fluo-8, p-CaMKII, and p-CaMKIV levels obtained from the images depicted in Fig. 3A. Fluorescence was quantified using Photoshop and normalized to their levels in D1 PAE, respectively. n=4. (**D**) Quantification of p-CaMKII and p-CaMKIV levels from Fig. 3B. Western blotting bands were quantified using ImageJ software and normalized to Tubulin levels. n=3. (**E**) RNAi knockdown efficiency of *RyR1* and *RyR2*. n=4. (**F**) Quantification of Fluo-8 levels obtained from the images depicted in Fig. 3C. Fluorescence was quantified using Photoshop and normalized to control. n=4. (**G**) Quantification of p-CaMKII and Jhamt levels from Fig. 3D. Western blotting bands were quantified using ImageJ software and normalized to Tubulin levels. n=3. (**H-I**) Immunofluorescence and quantification of p-CaMKII and Jhamt levels following knockdown of *RyR1* and *RyR2* (H), as well as treatments with inhibitors Dantrolene, CaMKII-IN-1 and KN-62 (I). Fluorescence was quantified using Photoshop and normalized to control. n=4. (**J**) The morphology of the ovaries upon blocking the release of Ca^2+^ from ER, disrupting calcium signaling, and rescue with methoprene (JH mimic). Scale bars: 20 μm (H, I), 5 mm (J, upper); 2 mm (J, lower). Data are mean ± SEM. Different letters indicate statistically significant differences (*P*<0.05). **P*<0.05, ***P*<0.01, ****P*<0.001.


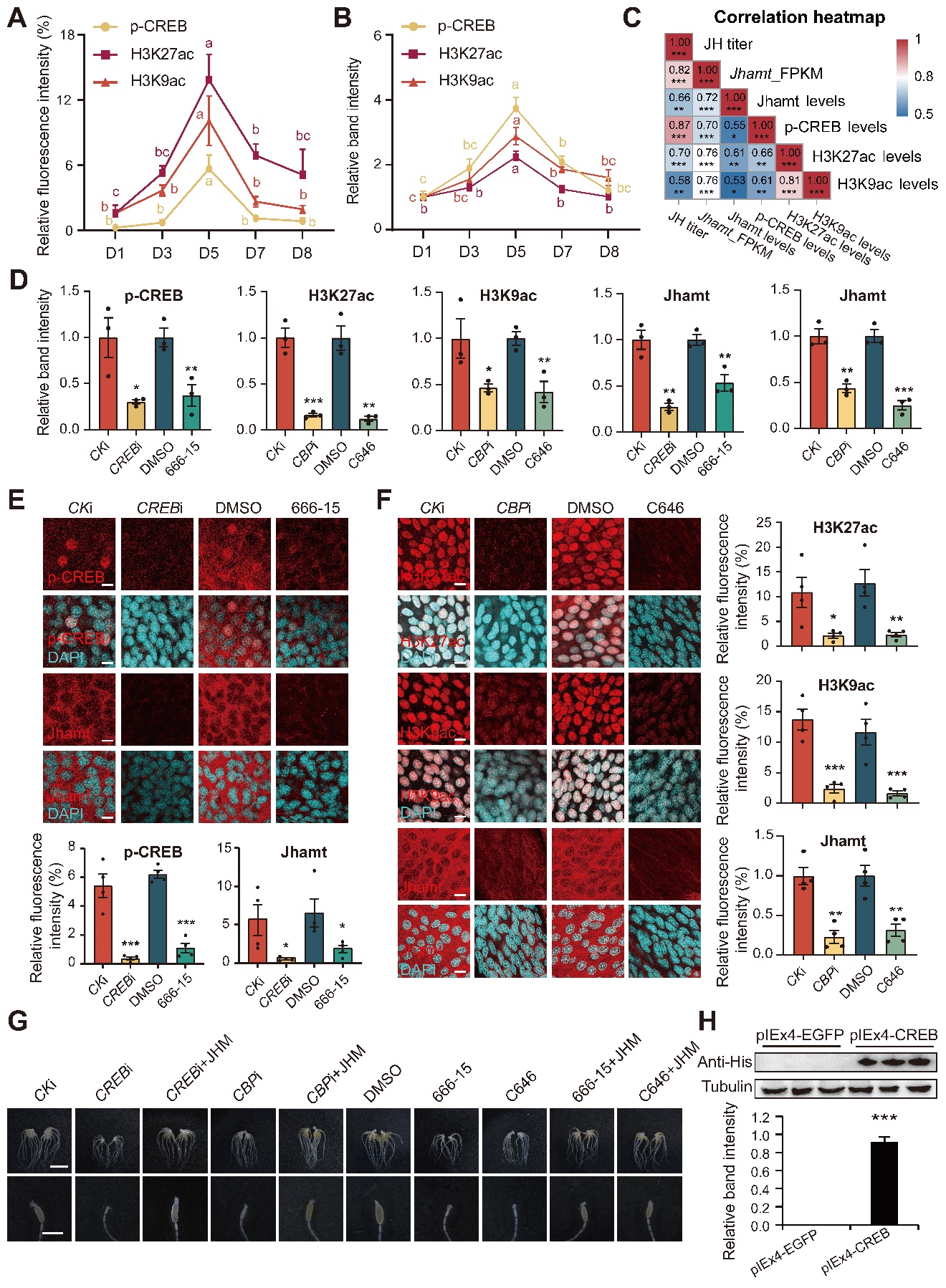


**Figure S4. CREB-CBP transcriptional activity depends on calcium signaling and regulates JH biosynthesis and female reproduction. Related to Figure 4.** (**A**) Quantification of p-CREB, H3K27ac and H3K9ac levels obtained from the images depicted in Fig. 4A. Immunofluorescence was quantified using Photoshop and normalized to their levels in D1 PAE, respectively. n=4. (**B**) Quantification of p-CREB, H3K27ac and H3K9ac levels from Fig. 4B. Western blotting bands were quantified using ImageJ software and normalized to Tubulin levels. n=3. (**C**) Correlation analysis p-CREB, H3K27ac and H3K9ac levels in relation to indicators reflecting JH biosynthesis. Spearman correlation analysis conducted utilizing the online platform Omicshare. (**D**) Quantification of p-CREB, H3K27ac, H3K9ac and Jhamt levels from Fig. 4C. Western blotting bands were quantified using ImageJ software and normalized to Tubulin levels. n=3. (**E**) Immunofluorescence and quantification of p-CREB and Jhamt levels following RNAi knockdown of *CREB*, as well as treatment with inhibitor 666-15. Fluorescence was quantified using Photoshop and normalized to control. n=4. (**F**) Immunofluorescence and quantification of H3K27ac, H3K9ac and Jhamt levels following knockdown of *CBP*, as well as treatment with inhibitor C646. Fluorescence was quantified using Photoshop and normalized to control. n=4. (**G**) The morphology of the ovaries following knockdown of *CREB* and *CBP*, and rescue with methoprene (JH mimic). (**H**) Successful overexpression of CREB protein in *Drosophila* KC cells confirmed by western blotting. Scale bars: 20 μm (E, F), 5 mm (G, upper); 2 mm (G, lower). Data are mean ± SEM. Different letters indicate statistically significant differences (*P*<0.05). **P*<0.05, ***P*<0.01, ****P*<0.001.


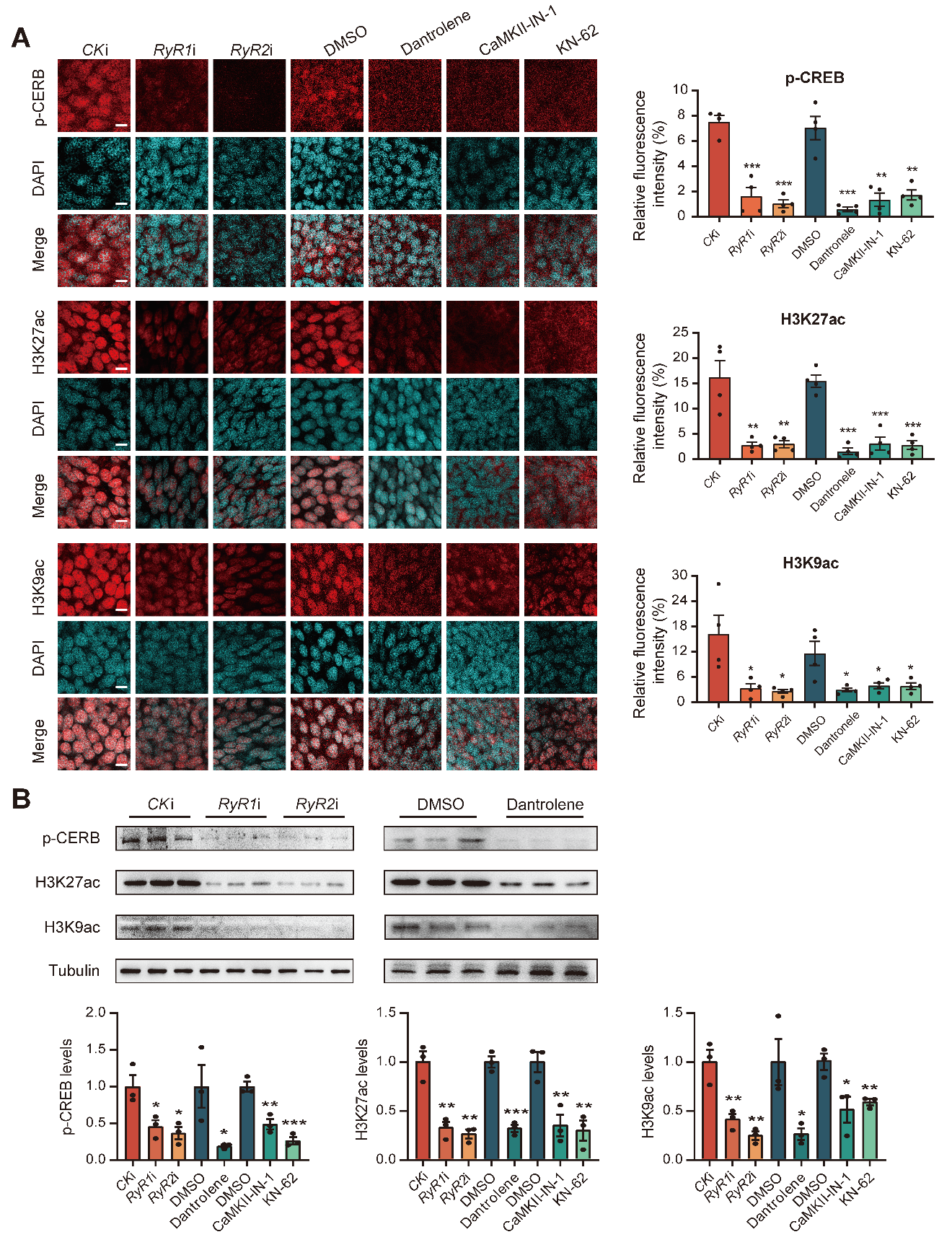


**Figure S5. RyR-mediated calcium signaling promotes p-CREB and H3K27ac and H3K9ac levels. Related to Figure 4.** (**A**) Immunofluorescence detection and quantification of p-CREB, H3K27ac and H3K9ac levels following dirsuption of calcium signaling. Fluorescence was quantified using Photoshop and normalized to control. n=4. (**B**) Western blotting detection and quantification of p-CREB, H3K27ac and H3K9ac levels following disruption of calcium signaling. Western blotting bands were quantified using ImageJ software and normalized to Tubulin levels. n=3. Scale bars: 20 μm (A). Data are mean ± SEM. **P*<0.05, ***P*<0.01, ****P*<0.001.


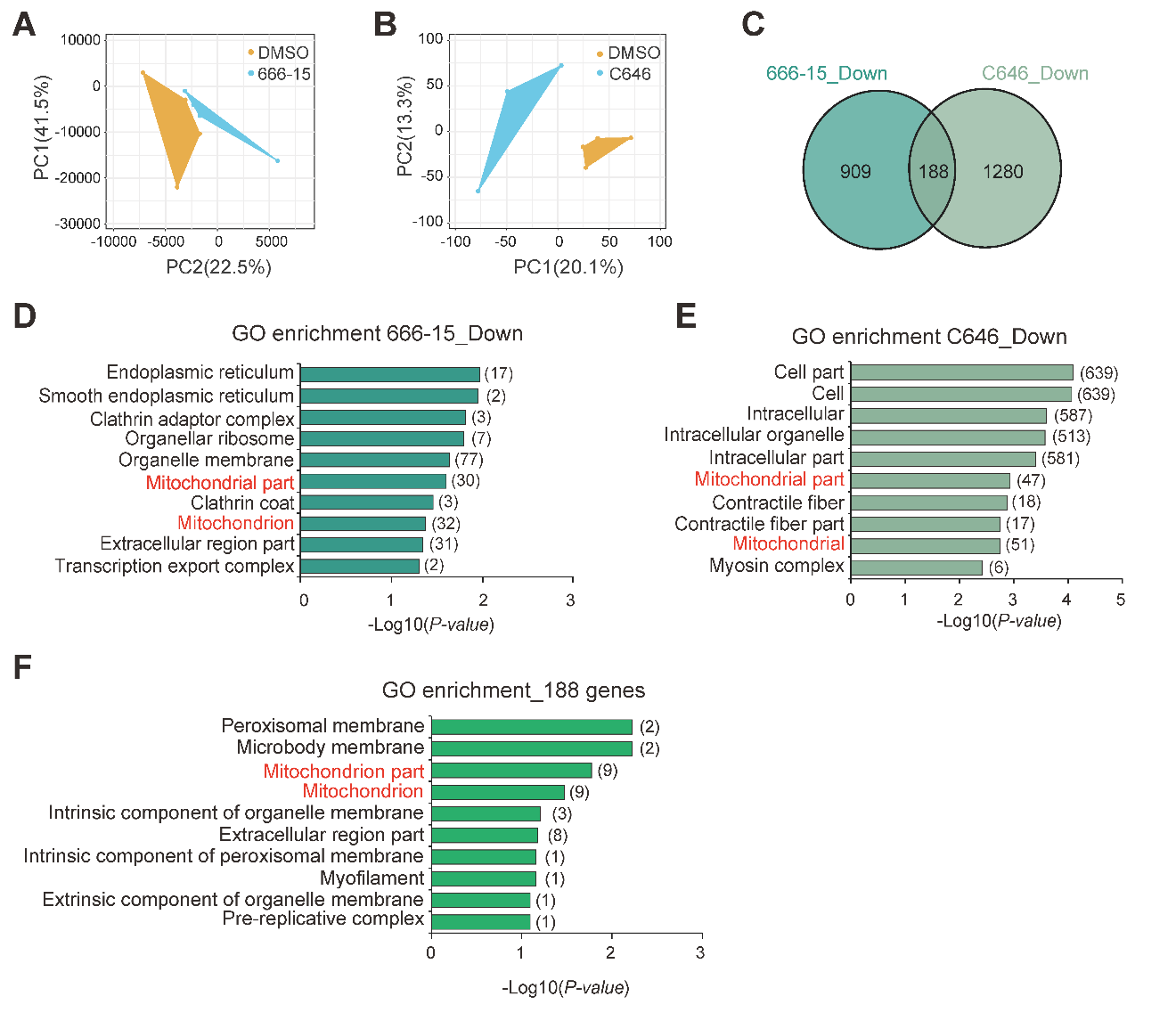


**Figure S6. Transcriptome analysis of differentially expressed genes following disruption of CREB-CBP. Related to Figure 4.** (**A and B**) PCA of differentially expressed genes following treatment with 666-15 (to block CREB) (A) and C646 (to block CBP) (B). (**C**) Venn diagram analysis of 1097 downregulated genes following treatment with 666-15 and 1468 downregulated genes following treatment with C646. A total of 188 genes overlap between their downregulated genes. (**D and E**) GO enrichment analysis of cellular component associated with 1097 downregulated genes in response to *CREB* knockdown by treatment with inhibitor 666-15 (D) and 1468 downregulated genes in response to *CBP* knockdown by treatment with inhibitor C646 (E). (**F**) GO enrichment analysis of cellular component associated with 188 overlapping genes from (C). Fold change>1.4, *P*-value<0.3. n=4. The horizontal axis represents the significance of differences in enriched gene sets or signaling pathways. The number adjacent to each bar on the chart indicates the total count of genes contained within a specific gene set or pathway. n=4.


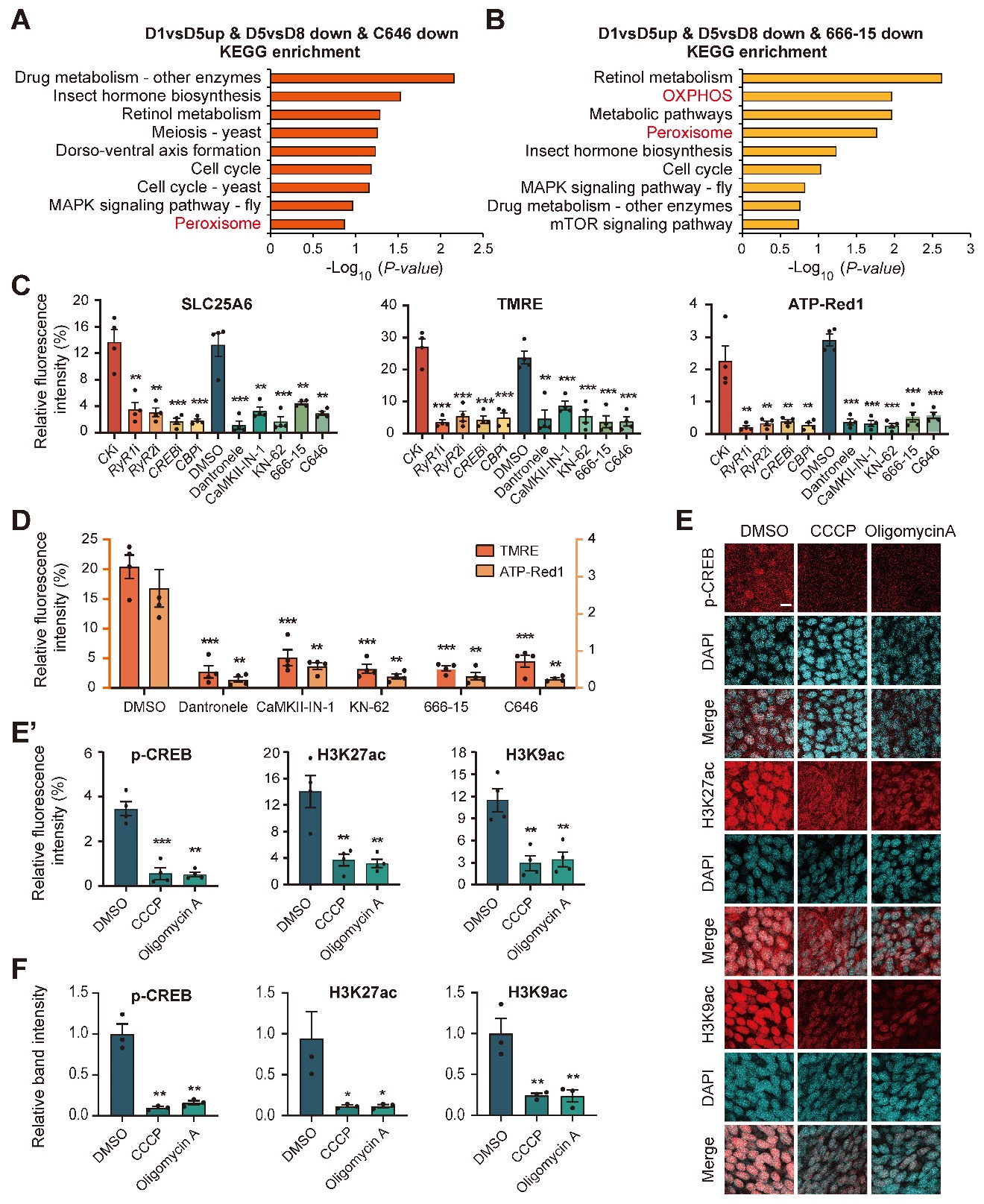


**Figure S7. Mitochondrial activity and CREB-CBP transcriptional activity boost each other.** **Related to Figure 5.** (**A**) KEGG enrichment analysis on the 106 overlapping genes that exhibited upregulation on D5 compared to D1 and D8, and downregulation following treatment with C646. The horizontal axis represents the significance of differences in enriched gene sets or signaling pathways. Fold change>1.4, *P*-value<0.3. n=3 or 4. (**B**) KEGG enrichment of 153 overlapped genes overlapped in the conditions of upregulation on D5 compared to D1 and D8, and downregulation upon treatment with C646. (**C**) Quantification of SLC25A6, TMRE, and ATP Red1 levels obtained from the images depicted in Fig. 5D. Fluorescence was quantified using Photoshop and normalized to control. n=4. (**D**) Quantification of TMRE and ATP Red1 levels obtained from the images depicted in Fig. 5E. Fluorescence was quantified using Photoshop and normalized to control. n=4. (**E and E’**) Immunofluorescence and quantification of p-CREB, H3K27ac, and H3K9ac levels following disruption of mitochondrial metabolic activity. Fluorescence was quantified using Photoshop and normalized to control. n=4. (**F**) Quantification of p-CREB, H3K27ac, and H3K9ac levels from Fig. 5F. Western blotting bands were quantified using ImageJ software and normalized to Tubulin levels. n=3. Scale bars: 20 μm (C). n=3 or 4. Data are mean ± SEM. **P*<0.05, ***P*<0.01, ****P*<0.001.


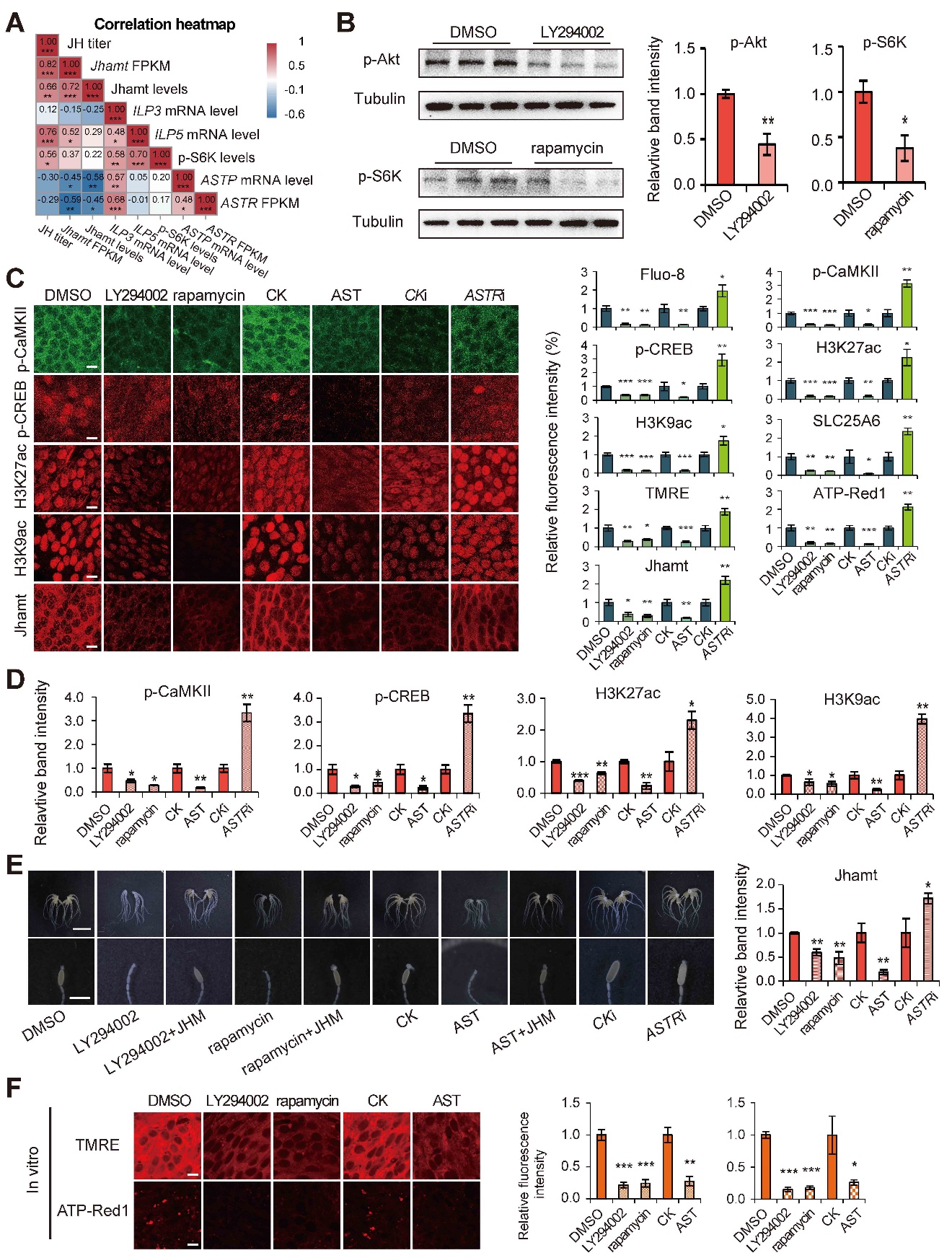


**Figure S8. Insulin and AST antagonistically regulate calcium signaling and mitochondrial activity. Related to Figure 6.** (**A**) Correlation analysis indicators reflecting IIS-TORC1 signaling in relation to those of reflecting JH biosynthesis. Spearman correlation analysis was conducted utilizing the online platform Omicshare. (**B**) Detection of p-Akt and p-S6K following treatment with inhibitors LY294002 and rapamycin, respectively. Western blotting bands were quantified using ImageJ software and normalized to Tubulin levels. n=3. (**C**) Immunofluorescence and quantification of p-CaMKII, p-CREB, H3K27ac, H3K9ac, and Jhamt levels following disruption of IIS-TORC1 and AST signaling. Quantification of Fluo-8, SLC25A6, TMRE and ATP Red1 levels obtained from the images depicted in Fig. 6D was also included. Fluorescence was quantified using Photoshop and normalized to control. n=4. (**D**) Quantification of p-CaMII, p-CREB, H3K27ac and H3K9ac levels from Fig. 6E. Western blotting bands were quantified using ImageJ software and normalized to Tubulin levels. n=3. (**E**) The morphology of the ovaries upon treatment with LY294002, rapamycin, AST, and *ASTR* dsRNA, as well as a rescue experiment with methoprene (JH mimic). (**F**) Fluorescence and quantification of TMRE and ATP Red1 levels following in vitro treatments with inhibitors CCCP and oligomycin A, as well as AST. Fluorescence was quantified using Photoshop and normalized to control. n=4. Scale bars: 20 μm (C, F), 5 mm (E, upper); 2 mm (E, lower). Data are mean ± SEM. **P*<0.05, ***P*<0.01, ****P*<0.001.


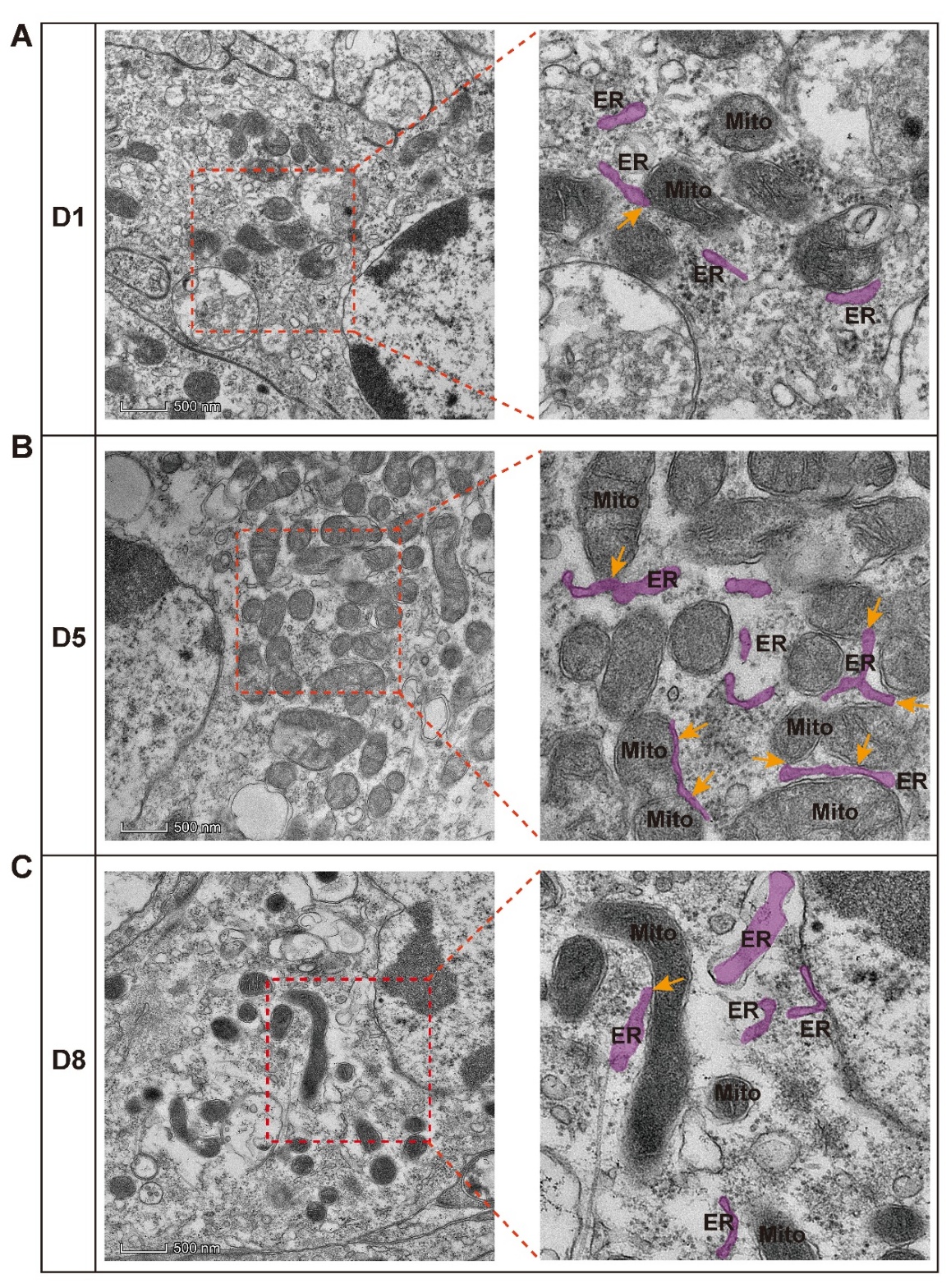


**Figure S9. Observations of physical contacts of ER and mitochondria in CA cells.** Transmission electron microscopy (TEM) of CA tissues on D1 (**A**), D5 (**B**) and D8 (**C**) during the first gonadotrophic cycle in adult female cockroaches. Detailed magnifications of the sections in the red boxes highlighting ER and mitochondria contact are shown in the right panels. The ER is stained with purple. Yellow arrows indicate ER-mitochondria contact sites. D5 shows more ER-mitochondria contact sites compared to D1 and D8. ER: endoplasmic reticulum. Mito: mitochondrion. Scale bars: 500 nm.


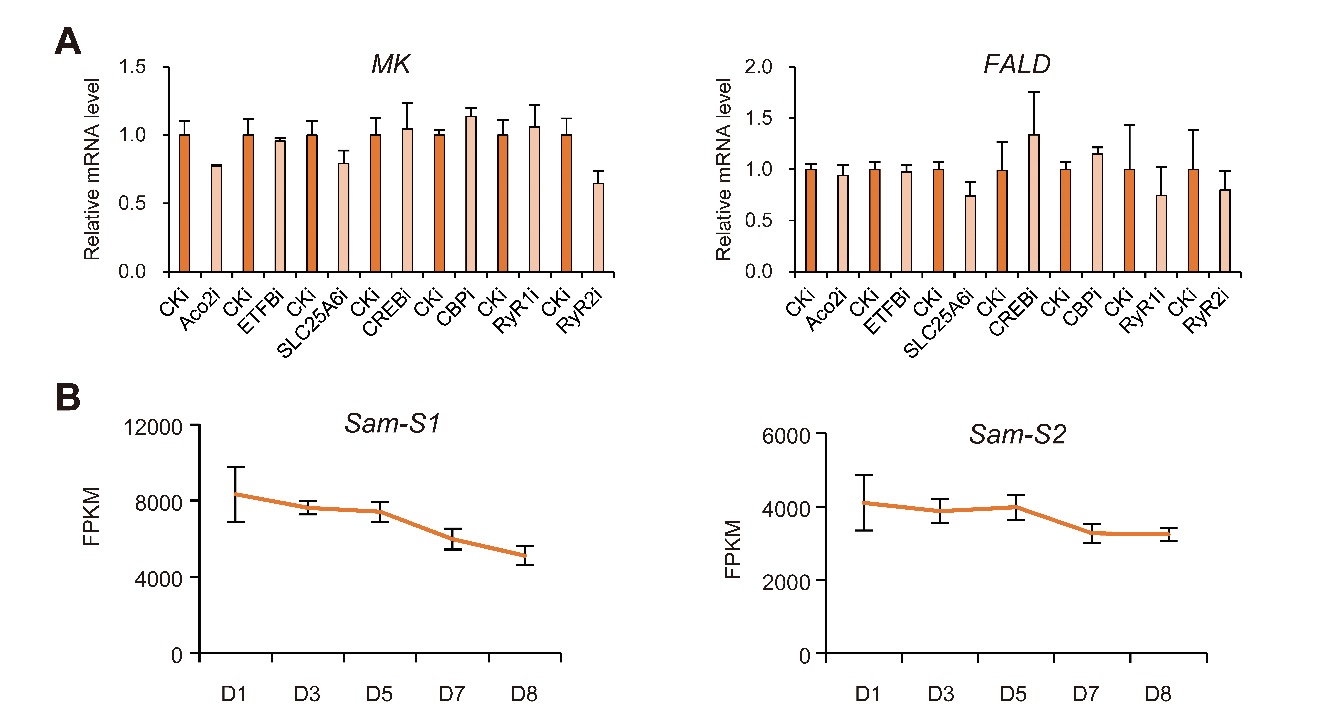


**Figure S10. The expression of non-fluctuating genes involved in JH biosynthetic pathway.** (**A**) The expression of *MK* and *FALD* following disruption of mitochondrial activity, CREB-CBP and calcium signaling. n=4. (**B**) The expression patterns of *Sam-Ss* during the first gonadotrophic cycle. The data was obtained from the CA transcriptome analysis in Figure 1. n=3. Data are mean ± SEM.

| **Table S1**  **Mitochondria-associated genes enriched in Fig. 5A.**   \| **id** \| **Mitochondria-associated genes** \| **Process** \| \| --- \| --- \| --- \| \| PaOGS02710 \| *slgA* \| Proline metabolism \| \| PaOGS04848 \| *PRDX3* \| ROS detoxification \| \| PaOGS17789 \| *Got1* \| Methionine salvage pathway \| \| PaOGS18778 \| *flr* \| actin disassembly \| \| PaOGS29228 \| *SLC25A6* \| ATP transport \| \| PaOGS29358 \| *CG12262* \| Fatty acid degradation \| \| PaOGS30806 \| *T05H10.6* \| TCA cycle \| \| PaOGS30855 \| *Hmgcll1* \| Valine, leucine and isoleucine degradation \|   **Table S2**  **Mitochondria-associated genes enriched in Fig. 5B.**   \| **id** \| **Mitochondria-associated genes** \| **Process** \| \| --- \| --- \| --- \| \| PaOGS01504 \| *SdhB* \| TCA cycle \| \| PaOGS12749 \| *Aco2* \| TCA cycle \| \| PaOGS05130 \| *C1QBP* \| Oxidative phosphorylative \| \| PaOGS21590 \| *NDUFS8* \| Oxidative phosphorylative \| \| PaOGS34384 \| *ETFB* \| Oxidative phosphorylation \| \| PaOGS29358 \| *CG12262* \| Fatty acid degradation \| \| PaOGS29359 \| *CG12262* \| Fatty acid degradation \| \| PaOGS35899 \| *scu* \| Fatty acid degradation \| \| PaOGS36662 \| *BCKDHB* \| Valine, leucine and isoleucine degradation \| \| PaOGS36663 \| *BCKDHB* \| Valine, leucine and isoleucine degradation \| \| PaOGS37265 \| *MCCC1* \| Valine, leucine and isoleucine degradation \| \| PaOGS01505 \| *EF-G2* \| Mitochondrial biogenesis \| \| PaOGS01656 \| *Tufm* \| Mitochondrial biogenesis \| \| PaOGS02710 \| *slgA* \| Proline metabolism \| \| PaOGS09568 \| *Coq3* \| Ubiquinone biosynthesis \| \| PaOGS36435 \| *flr* \| actin disassembly \|   **Table S3**  **Prediction of CRE in the promoter regions of JH biosynthetic genes and mitochondria-associated genes** | | | | |
| --- | --- | --- | --- | --- | --- | --- | --- | --- | --- | --- | --- | --- | --- | --- | --- | --- | --- | --- | --- | --- | --- | --- | --- | --- | --- | --- | --- | --- | --- | --- | --- | --- | --- | --- | --- | --- | --- | --- | --- | --- | --- | --- | --- | --- | --- | --- | --- | --- | --- | --- | --- | --- | --- | --- | --- | --- | --- | --- | --- | --- | --- | --- | --- | --- | --- | --- | --- | --- | --- | --- | --- | --- | --- | --- | --- | --- | --- | --- | --- | --- | --- | --- |
| **Promoters of genes** | **Matrix ID** | **Name** | **Score** | **Predicted CRE** |
| ***Thoil*** | MA0018.5 | MA0018.5.CREB1 | 14.63 | TGATGTCA |
|  | MA0018.4 | MA0018.4.CREB1 | 12.82 | CCTTGATGTCAGC |
|  | MA0018.5 | MA0018.5.CREB1 | 9.30 | TAATGTCA |
|  | MA0018.3 | MA0018.3.CREB1 | 10.36 | CTTGATGTCAGC |
|  | MA0018.2 | MA0018.2.CREB1 | 8.00 | TGATGTCA |
|  | MA0018.4 | MA0018.4.CREB1 | 9.75 | ATGTAATGTCAGT |
|  | MA0018.1 | MA0018.1.CREB1 | 8.02 | CTTGATCACGTT |
|  | MA0018.1 | MA0018.1.CREB1 | 8.02 | TACGATTACGTA |
|  | MA0018.5 | MA0018.5.CREB1 | 7.38 | TGATGAAA |
|  | MA0018.3 | MA0018.3.CREB1 | 7.46 | TACGACATCACA |
|  | MA0018.2 | MA0018.2.CREB1 | 6.58 | CGACATCA |
| ***HMGS*** | MA0018.4 | MA0018.4.CREB1 | 11.07 | ATAAGATGTCATA |
|  | MA0018.1 | MA0018.1.CREB1 | 9.63 | CAGAATGACGCA |
|  | MA0018.5 | MA0018.5.CREB1 | 9.38 | AGATGTCA |
|  | MA0018.5 | MA0018.5.CREB1 | 8.99 | TGATATCA |
|  | MA0018.2 | MA0018.2.CREB1 | 7.25 | TGACGCAT |
|  | MA0018.4 | MA0018.4.CREB1 | 7.99 | ATTTGATATCAGT |
|  | MA2209.1 | MA2209.1.CrebA | 9.06 | TGCCACATTTCA |
|  | MA0018.3 | MA0018.3.CREB1 | 7.20 | TAAGATGTCATA |
|  | MA0018.3 | MA0018.3.CREB1 | 7.04 | TTTGATATCAGT |
|  | MA0018.1 | MA0018.1.CREB1 | 6.57 | CTGAGTGGCGTG |
| ***MK*** | MA0018.5 | MA0018.5.CREB1 | 9.30 | TAATGTCA |
|  | MA0018.4 | MA0018.4.CREB1 | 8.27 | TACTAATGTCAAT |
|  | MA0018.1 | MA0018.1.CREB1 | 7.06 | TTGGATGACATT |
|  | MA0018.5 | MA0018.5.CREB1 | 6.37 | TGCTGACA |
|  | MA0018.2 | MA0018.2.CREB1 | 6.39 | TGACCTAA |
|  | MA1849.2 | MA1849.2.Creb1 | 6.25 | ATGACCTAAT |
|  | MA1849.2 | MA1849.2.Creb1 | 6.07 | ATTAAGTCAC |
|  | MA0018.3 | MA0018.3.CREB1 | 6.84 | AATGACCTAATC |
| ***PMK*** | MA0018.2 | MA0018.2.CREB1 | 9.47 | TGACGTGA |
|  | MA1849.2 | MA1849.2.Creb1 | 12.01 | ATCACGTCAC |
|  | MA0018.2 | MA0018.2.CREB1 | 9.00 | TCACGTCA |
|  | MA0018.3 | MA0018.3.CREB1 | 10.72 | CATCACGTCACC |
|  | MA0018.5 | MA0018.5.CREB1 | 9.30 | TAATGTCA |
|  | MA1849.1 | MA1849.1.Creb1 | 11.55 | ACAACCATCACGTCACCCAGCT |
|  | MA0018.1 | MA0018.1.CREB1 | 8.53 | AATGTTGACGTG |
|  | MA0018.4 | MA0018.4.CREB1 | 9.24 | GCATAATGTCATG |
|  | MA0018.5 | MA0018.5.CREB1 | 7.69 | TCACGTCA |
|  | MA0018.5 | MA0018.5.CREB1 | 7.63 | TGACGTGA |
|  | MA0018.4 | MA0018.4.CREB1 | 8.64 | AGGTTATGACATT |
|  | MA0018.5 | MA0018.5.CREB1 | 7.12 | TTATGACA |
|  | MA0018.2 | MA0018.2.CREB1 | 6.91 | TGACATGA |
|  | MA0018.4 | MA0018.4.CREB1 | 7.91 | CCATCACGTCACC |
|  | MA0018.3 | MA0018.3.CREB1 | 7.21 | GTTGACGTGAAC |
|  | MA0018.2 | MA0018.2.CREB1 | 6.44 | TCACATCA |
|  | MA0018.3 | MA0018.3.CREB1 | 6.77 | ACTGACATGAGC |
| ***PMMD*** | MA0018.4 | MA0018.4.CREB1 | 11.12 | TGGTGATCTCATT |
|  | MA0018.4 | MA0018.4.CREB1 | 9.93 | TGTTGATGTGATC |
|  | MA0018.5 | MA0018.5.CREB1 | 9.63 | TGATGTGA |
|  | MA0018.5 | MA0018.5.CREB1 | 9.39 | TGATCTCA |
|  | MA0018.3 | MA0018.3.CREB1 | 9.29 | GGTGATCTCATT |
|  | MA0018.3 | MA0018.3.CREB1 | 9.16 | TCTTACGTCAGT |
|  | MA0018.1 | MA0018.1.CREB1 | 8.29 | CCTGATCACGTT |
|  | MA0018.1 | MA0018.1.CREB1 | 8.12 | CCTGTTCACGTG |
|  | MA0018.5 | MA0018.5.CREB1 | 8.05 | TTACGTCA |
|  | MA0018.1 | MA0018.1.CREB1 | 7.94 | CTTGATGATGTT |
|  | MA1849.1 | MA1849.1.Creb1 | 7.91 | GACCTTCTTACGTCAGTATTTT |
|  | MA0018.2 | MA0018.2.CREB1 | 7.86 | TGACCTCA |
|  | MA0018.2 | MA0018.2.CREB1 | 7.86 | TTACGTCA |
|  | MA0018.2 | MA0018.2.CREB1 | 7.83 | TGACGTGT |
|  | MA0018.3 | MA0018.3.CREB1 | 7.64 | TTTGACCTCAAG |
|  | MA0018.4 | MA0018.4.CREB1 | 7.62 | TTCTTACGTCAGT |
|  | MA1849.2 | MA1849.2.Creb1 | 7.44 | ATTACGTGAC |
|  | MA0018.5 | MA0018.5.CREB1 | 7.39 | TGACCTCA |
|  | MA0018.1 | MA0018.1.CREB1 | 7.09 | TTTGTTGATGTG |
|  | MA0018.2 | MA0018.2.CREB1 | 6.91 | TCACGTTA |
|  | MA0018.5 | MA0018.5.CREB1 | 6.37 | TAATGACA |
|  | MA1849.2 | MA1849.2.Creb1 | 6.32 | CTTACGTCAG |
|  | MA1849.2 | MA1849.2.Creb1 | 6.20 | ATCACGTTAT |
|  | MA0018.5 | MA0018.5.CREB1 | 6.19 | TGAGTTCA |
| ***IPPI*** | MA0018.5 | MA0018.5.CREB1 | 9.34 | TGATTTCA |
|  | MA0018.5 | MA0018.5.CREB1 | 8.99 | TGATATCA |
|  | MA0018.4 | MA0018.4.CREB1 | 10.03 | TCATGATTTCACC |
|  | MA0018.4 | MA0018.4.CREB1 | 9.70 | TTATGATATCAGC |
|  | MA0018.3 | MA0018.3.CREB1 | 9.53 | TATGATATCAGC |
|  | MA0018.1 | MA0018.1.CREB1 | 8.17 | TTTTTTGACGTA |
|  | MA0018.4 | MA0018.4.CREB1 | 8.05 | AGATGATGATATC |
|  | MA0018.3 | MA0018.3.CREB1 | 8.00 | CATGATTTCACC |
|  | MA0018.3 | MA0018.3.CREB1 | 7.85 | GGTTACATCATG |
|  | MA0018.2 | MA0018.2.CREB1 | 6.69 | TGACGTAC |
|  | MA0018.5 | MA0018.5.CREB1 | 6.48 | TGATGATA |
|  | MA0018.5 | MA0018.5.CREB1 | 6.37 | TGCTGACA |
|  | MA1849.2 | MA1849.2.Creb1 | 6.42 | GTTACATCAT |
| ***FPPS*** | MA0018.5 | MA0018.5.CREB1 | 7.63 | TGACGTGA |
|  | MA2209.1 | MA2209.1.CrebA | 10.75 | TCACACGTGGCG |
|  | MA0018.5 | MA0018.5.CREB1 | 6.48 | TGATGATA |
|  | MA0018.5 | MA0018.5.CREB1 | 6.37 | TAATGACA |
|  | MA1849.2 | MA1849.2.Creb1 | 5.94 | ACGACGTCTT |
|  | MA0018.5 | MA0018.5.CREB1 | 5.87 | TGAGGTCT |
| ***FOLD*** | MA1849.2 | MA1849.2.Creb1 | 8.01 | GTTACGTAAT |
|  | MA1849.1 | MA1849.1.Creb1 | 7.91 | GAGATCGTGACGTCCAAGTATA |
|  | MA0018.5 | MA0018.5.CREB1 | 7.12 | TTATGACA |
|  | MA1849.2 | MA1849.2.Creb1 | 6.99 | GTGACGTCCA |
|  | MA0018.5 | MA0018.5.CREB1 | 6.51 | TGACGTCC |
| ***FALD*** | - | - | - | - |
| ***Aco2*** | MA2210.1 | MA2210.1.CrebB | 7.75 | GTTAAGTCAT |
|  | MA1849.2 | MA1849.2.Creb1 | 6.07 | GTTAAGTCAT |
|  | MA2210.1 | MA2210.1.CrebB | 7.30 | ATGACTTAAC |
|  | MA0018.2 | MA0018.2.CREB1 | 7.10 | TGAGGCAA |
|  | MA0018.1 | MA0018.1.CREB1 | 7.09 | TTTGTGGACGTG |
|  | MA0018.4 | MA0018.4.CREB1 | 7.38 | ATATGATTACATG |
|  | MA0018.5 | MA0018.5.CREB1 | 6.41 | TGATTACA |
|  | MA0018.2 | MA0018.2.CREB1 | 6.39 | TGACTTAA |
|  | MA1849.2 | MA1849.2.Creb1 | 6.07 | ATGACTTAAC |
| ***ETFB*** | MA0018.2 | MA0018.2.CREB1 | 9.00 | TGACATCA |
|  | MA0018.5 | MA0018.5.CREB1 | 9.67 | TGTTGTCA |
|  | MA0018.1 | MA0018.1.CREB1 | 9.38 | CTGGGTGACATC |
|  | MA0018.4 | MA0018.4.CREB1 | 10.45 | CTGTGTTGTCACA |
|  | MA0018.3 | MA0018.3.CREB1 | 10.35 | GCTGACATCAAT |
|  | MA0018.1 | MA0018.1.CREB1 | 8.89 | TTTGGTGACATC |
|  | MA0018.2 | MA0018.2.CREB1 | 7.36 | TGACATCT |
|  | MA0018.2 | MA0018.2.CREB1 | 7.36 | TGACATCT |
|  | MA0018.2 | MA0018.2.CREB1 | 7.36 | TGACATCT |
|  | MA0018.5 | MA0018.5.CREB1 | 7.12 | TTATGACA |
|  | MA0018.4 | MA0018.4.CREB1 | 8.09 | ATATTATGACATG |
|  | MA0018.5 | MA0018.5.CREB1 | 6.99 | TGACATCA |
|  | MA0018.4 | MA0018.4.CREB1 | 8.00 | TTGTGAGTTCATT |
|  | MA0018.3 | MA0018.3.CREB1 | 7.89 | GAAGACATCACA |
|  | MA0018.4 | MA0018.4.CREB1 | 7.90 | AGGTGCTGACATC |
|  | MA0018.3 | MA0018.3.CREB1 | 7.77 | GGTGACATCTTT |
|  | MA0018.5 | MA0018.5.CREB1 | 6.74 | TGTTGACA |
|  | MA0018.2 | MA0018.2.CREB1 | 6.66 | TGACCCCA |
|  | MA0018.1 | MA0018.1.CREB1 | 7.16 | GGTGCTGACATC |
|  | MA0018.5 | MA0018.5.CREB1 | 6.56 | TGGTGACA |
|  | MA0018.5 | MA0018.5.CREB1 | 6.37 | TGCTGACA |
|  | MA0018.3 | MA0018.3.CREB1 | 7.23 | TGTGTTGTCACA |
|  | MA0018.5 | MA0018.5.CREB1 | 6.19 | TGAGTTCA |
|  | MA0018.2 | MA0018.2.CREB1 | 6.33 | TGACACAA |
|  | MA0018.3 | MA0018.3.CREB1 | 6.99 | CATGACATCTTG |
|  | MA0018.2 | MA0018.2.CREB1 | 6.22 | TGACTTCT |
|  | MA0018.3 | MA0018.3.CREB1 | 6.76 | TGTGAGTTCATT |
|  | MA0018.4 | MA0018.4.CREB1 | 6.73 | ATTTGGTGACATC |
| ***SLC25A6*** | MA0018.5 | MA0018.5.CREB1 | 9.63 | TGATGTGA |
|  | MA0018.5 | MA0018.5.CREB1 | 9.41 | TGATGTTA |
|  | MA0018.5 | MA0018.5.CREB1 | 9.03 | TGATGTCT |
|  | MA0018.4 | MA0018.4.CREB1 | 9.73 | TGATGATGTGAAT |
|  | MA0018.1 | MA0018.1.CREB1 | 8.61 | CCTGATGATGTG |
|  | MA0018.5 | MA0018.5.CREB1 | 6.90 | TTAGGTCA |
|  | MA0018.4 | MA0018.4.CREB1 | 7.41 | GTCTGATGTCTAT |
|  | MA0018.4 | MA0018.4.CREB1 | 7.37 | ATATTATGTAATT |
|  | MA0018.5 | MA0018.5.CREB1 | 6.37 | TAATGACA |

**Table S4**

**Primers for qPCR**

| Gene | Forward primer | Reverse primer |
| --- | --- | --- |
| *actin* | TCCAGCTCACTGGAGAAATC | GGAGTTGTATGTGGTCTCGT |
| *SCL25A6* | CCCTTAGACTACGCCCGAAC | TCTTCACGATGCAGTCCACC |
| *Aco2* | GTGGCACGAGCCTACAAGAA | GTTTCATGAATGCGGGCGAA |
| *ETFB* | CACAGTCTCAGGAAACCCTCC | CAAGGTTTCATACTCTGGCCC |
| *HMGR* | AGCATGCTTGGAGATGCTTG | TGCTGACATGAGGGACAGTT |
| *Jhamt* | CCTTGACCGCATTCCACAAA | GCGCGTATGCGATAATGAGT |
| *Cyp15a1* | ATATGGACTCCGAGCACTGG | GCATTTCTCCAAGACAGCGT |
| *RyR1* | TGTCCGGACTTTGCTGGTTT | TACTTCTTGCTGCTTCGGCA |
| *RyR2* | TCACTCGTGGACTTGTTGGG | CCTCCACTGCAGCCATAACA |
| *CBP* | AGCAGGAGATTGACCCCGTA | GTGTGACTGTGTCTCCTGGG |
| *CREB* | TAGTGCAGTACGCTCAAGGG | CTTCAGCTATAATTGGCCCGC |
| *ILP3* | AACGGCACCTACTACACCAA | ATCCACACATCGTCGTCTGA |
| *ILP5* | ACCTACAAGAACGCGGGTAA | TTAGACCTGTCGGGCGATTT |
| *ASTP* | GCAAGCGTGATTACGACGAC | TGTAAGGCCTTGCCCTCTTG |
| *ASTR* | ATCGGCGGACTTCGTTATCA | TTCTGGATGAGCTGCAAGGA |
| *MK* | AGCTGGAGGGGGAGGATATG | CTGCCAAGTTCTGTCTCGGT |
| *FALD* | ACATATGCCTCCCCCTGGAA | ACTAGACCAGGACTGACGCT |

**Table S5**

**Primers for PCR**

| Gene | Forward primer | Reverse primer |
| --- | --- | --- |
| *SCL25A6* | GCTTCTGATCGGGAATACCA | CATCATACAGCACAAGCACC |
| *Aco2* | GACGATATTGCCAGTGAAGC | AGCAATAGACAGTGCCGTTA |
| *ETFB* | CTGGGCCAGAGTATGAAACC | ACTACCTCAATACGTGCTGC |
| *RyR1* | CCCGAGAGTGAAATCATGGA | CGCCACAAGATTTTGCACTA |
| *RyR2* | AAGTATGGAAGAAGGAGGCG | TTCGCCTCCTTCTTCCATAC |
| *CREB* | ATCAGCAGTCTGTCATCCAG | CCCTTGTACAGTTTCTCCCT |
| *CBP* | CAAGCAGCTTATTGCACTGT | AGCGTTGGAATTAAGTGTGC |
| *ASTR* | GTCACGTACACCTACTCCTC | CTGCTGCTGAATGGTTGAAG |
| *Cyp15a1* CDS | ATGTGGCTGCCTGTTCTGGGCAACC | TCATGTTCTTGGTACTAATTTCGC |

**Table S6**

**Oligonucleotide probes for EMSA**

| Name | Forward | Reverse |
| --- | --- | --- |
| WT probe | TGCTTCGGCATGTGAACATGAGGTCAGCAGCCGGCTGGTT | AACCAGCCGGCTGCTGACCTCATGTTCACATGCCGAAGCA |
| Mutant probe | TGCTTCGGCATGTGAACAGACAAGTGGCAGCCGGCTGGTT | AACCAGCCGGCTGCCACTTGTCTGTTCACATGCCGAAGCA |
| Cold probe | TGCTTCGGCATGTGAACATGAGGTCAGCAGCCGGCTGGTT | AACCAGCCGGCTGCTGACCTCATGTTCACATGCCGAAGCA |

**Materials and methods**

**Animals.** The American cockroach belongs to the insect order Blattodea and often serves as a model insect [1]. The cockroaches used in our experiments have been previously described and maintained under controlled laboratory conditions [2, 3]. We collected synchronized adult females and performed necessary procedures under CO_2_ anesthesia to ensure animal welfare. Tissues were collected in cockroach saline solution. Our experiments included at least three biological replicates with a minimum of 30 animals per replicate to ensure the reliability of the results.

**RNA-seq and assembly.** CA samples were dissected from adult female cockroaches and immediately flash frozen in liquid nitrogen to preserve RNA integrity. 60-80 pairs of CA were collected for each replicate. Samples were stored at -80°C until further processing. Total RNA was isolated using the Direct-zol RNA Miniprep Kit (R2072, Zymo Research) including a genomic DNA digestion step. Over 1 μg of RNA per sample was used for library preparation. The resulting cDNA libraries were sequenced on the Illumina NovaSeq6000 platform by Biomarker Technologies. After quality control and duplicate removal, clean reads were aligned to the *P. americana* reference genome [2] using Hisat2. Gene expression levels were calculated as fragments per kilobase of transcript per million mapped reads (FPKM). De novo assembly of clean reads into coding sequences was performed using Trinity with default parameters.

**Total RNA extraction and quantitative real-time PCR (qPCR).** CA samples were collected and rapidly frozen in liquid nitrogen to preserve RNA integrity, and then stored at -80°C until further processing. Total RNA was extracted from the CA using a Direct-zol RNA MiniPrep kit (R2072, Zymo Research) according to the manufacturer’s instructions. cDNA synthesis was performed using reverse transcriptase M-MLV (2641A, TaKaRa) according to the manufacturer’s protocol. Quantitative real-time PCR (qPCR) was carried out using Hieff® qPCR SYBR Green Master Mix (Low Rox Plus) (11202ES03, Yeasen) on the Applied Biosystems QuantStudio 6 Flex Real-Time PCR System (4485691, Thermo Fisher). The reference gene chosen for qPCR normalization was *actin* using the ΔΔCt method. Target genes expression levels were standardized to *actin*. Primer sequences used for qPCR can be found in Table S4.

**Transmission electron microscopy (TEM).** CA tissues were dissected and fixed in 2.5% glutaraldehyde at 4℃ overnight, followed by washing with PBS. Tissues were then fixed in a 1% osmium tetroxide solution for 3 hours, washed again in PBS, and stained overnight in 3% uranyl acetate. After washing with distilled water, the tissues were dehydrated in a graded series of ethanol. Tissues were then sequentially infiltrated with 1:1 and 1:3 ratios of acetone: resin for 12 hours each, and finally embedded in pure resin. Polymerization was performed at 70℃ for 2 hours. Samples were sliced using a UC7 ultramicrotome (Leica) and imaged using a Talos L120C transmission electron microscope (Thermo Fisher).

**RNAi knockdown.** Fragments ranging from 400-600 bp were amplified from target gene cDNA to generate templates for dsRNA synthesis. PCR primers containing T7 promoter sequences were used to amplify dsRNA from these templates. Double-stranded RNA (dsRNA) was synthesized using the T7 RiboMAX Express RNAi Kit (P1700, Promega) following the manufacturer’s instructions. A control dsRNA was synthesized (CK dsRNA) based on a 92 bp non-coding sequence from the pSTBlue-1 vector, which has been described previously [4]. dsRNA concentrations were measured by NanoDrop (Thermo Scientific) and 4 μg were injected into the abdomen of each cockroach on days 2 and 4 post adult eclosion (PAE). Control cockroaches received CK dsRNA injections on the same schedule. Tissues were collected for analysis after treatments. All PCR primers used for dsRNA synthesis are listed in Table S5.

**Injection with inhibitors and peptides.** 10 μg of dantrolene (sodium salt) (B6329, APExBIO) (RyR inhibitor), 10 μg of KN62 (GC14202, Glpbio) (CaMKII inhibitor), 10 μg of CaMKII-IN-1 (GC35601, Glpbio) (CaMKII inhibitor), 10 μg of CCCP (S6494, Selleck) (OXPHOS inhibitor), 3 μg Oligomycin A (S1478, Selleck) (OXPHOS inhibitor), 4ug of rapamycin (HY-10219, MCE) (TORC1 inhibitor), and 100 μg of synthetic neuropeptide AST (IGE Bio; Sequence: SPSGMQRLYGFGL-NH_2_) were injected into adult female cockroaches on day 2 and day 4 PAE. Control groups were injected with the corresponding volume of the solvent. Tissues were collected on day 5 PAE for further use.

**Rescue experiments.** For rescue experiments, 100 μg of methoprene was injected into the abdomen of each cockroach on days 2 and 4 post adult eclosion (PAE), immediately after RNAi knockdown or injection of inhibitors or AST peptides. Control groups were injected with the corresponding volume of the solvent. Tissues were collected on day 5 PAE for further use.

**Tissue imaging, cell staining, and confocal microscopy.** For RNAi, inhibitor and AST injection experiments, the ovaries were dissected on day 5 PAE and imaged with a Nikon DS-Ri2 camera and a Nikon SMZ25 microscope. Primary oocyte length was measured for each ovary using NIS-Elements BR 4.50.00 software (Nikon) [5].

CA tissues were dissected, fixed and permeabilized in a solution of 4% paraformaldehyde and 0.6% Triton X-100 for 1 hour. Afterward, they underwent three 15-minute washes in phosphate-buffered saline with Triton X-100 (PBT). Samples were then incubated overnight at 4°C with primary antibodies against Jhamt (1:100) [6] (a gift from Dr. Yu Bai in our laboratory), SLC25A6 (1:100, DF3742, Affinity), p-CaMKII (1:100, ab5683, abcam), p-CaMKIV (1:100, PA5-38435, Thermo), p-CREB (1:100, 9198, Cell Signaling Technology), H3K27ac (1:100, ab4729, abcam), H3K9ac (1:100, ab32129, abcam), or p-S6K (1:100, AF3228, Affinity), respectively. After a further three 15-minute washes in PBT, the samples were incubated for 2 hours at room temperature in a secondary antibody solution consisting of Goat Anti-Rabbit IgG H&L (Alexa Fluor® 488) (1:1000, ab150077, abcam) or Goat Anti-Rabbit IgG H&L (Alexa Fluor® 594) (1:1000, ab150080, abcam). For calcium ion, mitochondrial membrane potential, and ATP staining, CA samples were dissected and incubated with Fluo-8 AM (1:1000, ab142773, abcam), TMRE (1:1000, HY-D0985A, MCE), and ATP-red 1 (1:1000, HY-U00451, MCE) for 1 hour at room temperature, respectively. Confocal microscopy images were captured using an Olympus FluoView FV3000 confocal microscope and processed using FV31S-SW software (Olympus).

**Hemolymph collection and preparation.** Hemolymph samples were collected directly into salinized 1.8 mL vials placed on ice. A total of 100 μL of hemolymph was collected for each sample. To each sample, 100 μL of 50% methanol and 400 μL of hexane were added. The samples were then vortexed for 1 min and then centrifuged for 5 min at 2000 g at 4 °C. The upper organic phase was transferred to a new silanized vial and dried under nitrogen flow. The dried samples were then resolved in 50% methanol for LC-MS/MS analysis or stored at -20 °C until use.

**JH quantification.** The extracted JH was quantified by LC-MS/MS. Analysis was performed using a SCIEX QTRAP 4500 MD tandem mass spectrometer coupled with a Shimadzu Exion LC UHPLC system. A Waters BEH C18 column (130 Å, 1.7μm, 2.1 mm X 50 mm) was used for separation. The mobile phase consisted of water/acetonitrile/formic acid (solvent A: 98/2/0.1%; solvent B: 2/98/0.1%). The gradient elution raised solvent B from 10% to 85% over 10 minutes. Detection was in electrospray positive ion mode, with targeted quantification of JH by multiple reaction monitoring.

**In vitro JH biosynthesis assay.** JH biosynthesis assays using corpora allata-corpora cardiaca (CA-CC) complexes were conducted following the methods described in Ramirez et al [7], with modifications. CA-CC complexes were dissected as isolated glands from wild type females at day 4 PAE and incubated in 200 μL of Grace’s insect medium (11605094, Thermo Scientific). To investigate the influence of mitochondrial metabolism, 25 μM carbonyl cyanide m-chlorophenyl hydrazine (CCCP) or 5 μM oligomycin A were incorporated into the medium. For the assessment of calcium signaling, a range of CaCl_2_ concentrations (0-2 mM), 2 mM ethylenediaminetetraacetic acid (EDTA), 4 μM dantrolene, 5 μM CaMKII-IN-1, or 10 μM KN-62 were applied. The roles of CREB and CBP were probed using 1 μM 666-15 or 5 μM C646, respectively. Additionally, the involvement of the IIS-TORC1 and AST was examined with 10 μM LY294002, 25 μM rapamycin, or 10 μM AST, respectively. Control groups received an equivalent volume of solvent. The selection and application of these inhibitors and reagents are detailed in the preceding section titled "Injection with inhibitors and peptides."

To delineate the role of the IIS in signal transduction, we performed knockdown of *RyR1*, *RyR2*, *Aco2*, *ETFB*, and *SLC25A6* and then dissected CA-CC tissues and supplemented the culture medium with 10 μM bovine insulin (S12033, Shanghai Yuanye Bio-Technology). The incubations were carried out in a humid chamber in silanized 1.8 mL vials at 30 °C for 4 hours in the dark, with continuous gentle agitation. After incubation, the tissues were removed, and the medium samples were collected in new silanized vials. Then, 600 μL of hexane was added to each sample, which was vortexed for 1 minute and centrifuged at 2000 g for 5 minutes at 4 °C. The upper organic phase was transferred to a new silanized vial and dried under a nitrogen flow. The dried powder was dissolved in 50% methanol or stored at -20 °C until LC/MS-MS analysis.

**Bioinformatics analysis.** Gene ontology (GO) enrichment analysis, Kyoto Encyclopedia of Genes and Genomes (KEGG) enrichment analysi, gene set enrichment analysis (GSEA), and correlation analysis were conducted with the differentially expressed genes using the GO, KEGG, GSEA, and spearman’s correlation analysis program on the OmicShare website (https:// www. omics hare. com/ tools/ home/ soft/ getso ft. html) or Omicsmart website (https:// www. omics mart. com). The prediction of CREs was conducted using the JASPAR website (https://jaspar.elixir.no/analysis).

**Western blotting analysis.** Total proteins were extracted from CA tissues and KC cells transfected with pIEx4-CREB or pIEx4-EGFP, respectively. CA tissues or cells were homogenized in RIPA Lysis Buffer (P0013B, Beyotime) supplemented with 1% (v/v) phenylmethylsulfonyl fluoride. CA samples were collected and each replicate contains a minimum of 100 pairs of CA tissues. Protein concentration was determined using the Bradford protein assay kit (P0006, Beyotime). Each lane was loaded with 10 μg of protein extract, separated by 10% sodium dodecyl sulfate–polyacrylamide gel electrophoresis, and then transferred to polyvinylidene difluoride membranes (ISEQ00010, Millipore). Tubulin protein was used as a loading control. The membranes were incubated overnight at 4 °C with primary antibodies against Jhamt [6] (a gift from Dr. Yu Bai in our laboratory), p-CaMKII (ab5683, abcam), p-CaMKIV (PA5-38435, Thermo), p-CREB (9198, Cell Signaling Technology), H3K27ac (ab4729, abcam), H3K9ac (ab32129, abcam), p-Akt (9271, Cell Signaling Technology), p-S6K (AF3228, Affinity), His-Tag (12698S, Cell Signaling Technology), and Tubulin (AF1216, Beyotime), at dilutions of 1:5000, respectively. Immobilon Western Chemiluminescent HRP Substrate (Thermo, USA) was used for protein detection.

**Dual-luciferase reporter assay.** The complete coding sequence (CDS) of the transcription factor *CREB* was amplified from the American cockroach genome [2] and inserted into the Kpn I and Not I restriction sites of the pIEx4 vector (Promega) using the Hieff Clone Plus One Step Cloning Kit (10911ES20 Yeasen). Fragments of the *HMGR*, *Jhamt*, and *Cyp15a1* promoter regions were also amplified and subcloned into the Kpn I and Bgl II sites of the pGL3-basic vector (Promega) fused to the firefly luciferase reporter gene.

For transfection, the pGL3 reporter plasmids, the pIEX4-DsxM expression plasmid, and a reference pGL4.73[hRluc/SV40] vector were co-transfected into *Drosophila* Kc cells and cultured in 96-well plates at 27°C for 48 hours. A negative control was included using the pIEx4-EGFP vector. Successful expression of CREB was confirmed by western blotting. Luciferase activity was measured using the Dual Luciferase Reporter Gene Assay Kit II (RG029S, Beyotime) and a GloMax 96 Microplate Luminometer (Promega) [8]. This transfection process was repeated three times independently.

**Electrophoretic mobility shift assay (EMSA).** An EMSA was performed using a LightShift Chemiluminescent EMSA Kit (20148, Thermo Scientific). To overexpress CREB protein in KC cells, the pIEx4-CREB-His vector was transfected, and nuclear proteins were extracted using a Nuclear and Cytoplasmic Protein Extraction Kit (P0027, Beyotime). A specific fragment (341- ~ -302 nt) from the *Cyp15a1* promoter region was synthesized and labelled with 5-FAM at the 5’ terminus. Double-stranded probes were generated by annealing DNA oligonucleotides at 95°C for 1 min. Binding reactions were carried out at room temperature in a 20 μL reaction system comprising 1× binding buffer, 2.5% glycerol, 5 mM MgCl_2_, 0.05% NP-40, and 1 μg of poly(dI·dC). For competition assays, unlabelled cold probes and WT or mutant probes were added to the binding reaction in 100- or 200-fold molar excess into the binding reaction. In the super-shift assay, His antibody was pre-incubated with nuclear protein extracts for 2 hours at 4 °C before adding the WT probe. All reactions were then incubated for 20 minutes at room temperature and separated by 6% native acrylamide gel electrophoresis in 0.5× TBE buffer on ice at 100 volts for 1.5 hours. Images were captured using a Tanon-5500 Chemiluminescent Imaging System (Tanon). The oligonucleotide probes used in this assay are provided in Supplementary Table S6.

**Data analysis.** Statistical analyses were conducted using IBM SPSS Statistics v.19.0 software. All error bars represent the standard error of the mean (SEM). Student's t-test was used to determine significant differences between two independent groups. Statistical analysis of developmental profiles in JH titers, genes, proteins, and dye staining was conducted using one-way ANOVA and post-hoc Duncan multiple range test. Statistical data were plotted with GraphPad Prism v8.0.2 and figures were formatted using Adobe Illustrator CC. Schematic view was created by Figdraw, an online platform (https://www.figdraw.com/static/index.html#/), and formatted using Adobe Illustrator CC.

**References**

1. Ren C, Chen N, Li S. Harnessing “Little Mighty” cockroaches:Pest management and beneficial utilization. *The Innovation* 2023; **4**: 100531.

2. Li S, Zhu S, Jia Q *et al.* The genomic and functional landscapes of developmental plasticity in the American cockroach. *Nat Commun* 2018; **9**: 1008.

3. Zhu S, Liu F, Zeng H *et al.* Insulin/IGF signaling and TORC1 promote vitellogenesis via inducing juvenile hormone biosynthesis in the American cockroach. *Development* 2020; **147**.

4. Zhu S, Chen X, Xia S *et al.* Hexamerin and allergen are required for female reproduction in the American cockroach, *Periplaneta americana*. *Insect Sci* 2023.

5. Liu F, Cui Y, Lu H *et al.* Myofilaments promote wing expansion and maintain genitalia morphology in the American cockroach, *Periplaneta americana*. *Insect Mol Biol* 2023; **32**: 46-55.

6. Li Z, Zhou C, Chen Y *et al.* Egfr signaling promotes juvenile hormone biosynthesis in the German cockroach. *BMC Biol* 2022; **20**: 278.

7. Ramirez CE, Nouzova M, Michalkova V *et al.* Common structural features facilitate the simultaneous identification and quantification of the five most common juvenile hormones by liquid chromatography-tandem mass spectrometry. *Insect Biochem Mol Biol* 2020; **116**: 103287.

8. Chen-Engerer HJ, Hartmann J, Karl RM *et al.* Two types of functionally distinct Ca(2+) stores in hippocampal neurons. *Nat Commun* 2019; **10**: 3223.

9. Zhao S, Deanhardt B, Barlow GT *et al.* Chromatin-based reprogramming of a courtship regulator by concurrent pheromone perception and hormone signaling. *Sci Adv* 2020; **6**: eaba6913.

10. Zhang XS, Wang ZH, Li WS *et al.* FoxO induces pupal diapause by decreasing TGFβ signaling. *Proc Natl Acad Sci U S A* 2022; **119**: e2210404119.
